# Have declines in the prevalence of young adult drinking in English-speaking high-income countries followed declines in youth drinking? A systematic review

**DOI:** 10.1080/09687637.2024.2335989

**Published:** 2024-05-22

**Authors:** Jessica Dunphy, Emma Vieira, Abigail K. Stevely, Michael Livingston, Rakhi Vashishtha, Kirsten Rivelin, John Holmes

**Affiliations:** a Sheffield Addictions Research Group, School of Medicine and Population Health, University of Sheffield, Sheffield, UK; b National Drug Research Institute, Curtin University, Perth, Australia; c Centre For Alcohol Policy Research, La Trobe University, Melbourne, Australia; d Department of Clinical Neurosciences, Karolinska Institutet, Stockholm, Sweden; e Health Services and Systems Research, Duke-NUS Medical School, Singapore

**Keywords:** Alcohol drinking, underage drinking, young people, adults, trends

## Abstract

**Background:**

Alcohol use in early adulthood is a significant public health concern. The prevalence of adolescent alcohol consumption has been declining in high-income English-speaking countries since the early 2000s. This review aims to examine whether this trend continues in young adulthood.

**Methods:**

We systematically searched Medline, PsycInfo and CINAHL and the grey literature. Eligible records reported the prevalence of alcohol consumption amongst 18–25-year-olds over a minimum three-year time frame in the United States (US), Canada, the United Kingdom, the Republic of Ireland, Australia and New Zealand. Results were described using narrative synthesis. Quality assessment was undertaken using the Joanna Briggs Institute Critical Appraisal Checklist for Prevalence Studies.

**Results and conclusion:**

Thirty-two records from 22 different surveys were included. The prevalence of consumption amongst young adults fell in Australia, Ireland, and the United Kingdom and was stable in New Zealand and Canada. In the US, there was evidence of a decline in the prevalence of drinking among under-21s, but results for adults over the minimum purchase age were mixed. The prevalence of alcohol consumption in young adults appears to be broadly declining. This could lead to reduced rates of alcohol-related harms in the future. Further high-quality multinational surveys may help to confirm this trend.

## Introduction

Adolescent and young adult alcohol use is a significant public health concern (Marshall, 2014; WHO, 2018). Initiation of alcohol use generally occurs during adolescence (Marshall, 2014; WHO, 2018) and peaks during young adulthood (Leggat et al., 2022; Patrick et al., 2016). Early initiation of alcohol is associated with alcohol use disorders in adulthood, which cause a significant burden of morbidity and mortality worldwide (Degenhardt et al., 2018; Rehm et al., 2009; WHO, 2018). In addition, adolescent alcohol use clusters with other issues in emerging adulthood such as academic problems, tobacco and illicit drug use, and risky sexual behavior (Meader et al., 2016; Whitaker et al., 2021).

Recent data from the United States (US), Canada, Australia, and many European countries, however, shows that the proportion of the adolescent population who consume any alcohol has declined, including a reduction in how often they are drinking and how many drinks they consume per session (Kraus et al., 2018; Oldham et al., 2018; Pape et al., 2018; Vashishtha et al., 2021). The extent and timing of this decline varies between countries, with the earliest and largest declines seen in English-speaking countries and northern Europe (Kraus et al., 2018; Vashishtha et al., 2021). Despite a growing body of research, the reasons for this decline are not yet clear, with various factors suggested to have played a role (De Looze et al., 2019; Vashishtha et al., 2020, 2021). As adolescents mature into young adults and often look to move out of the parental home and start a career, their attitudes, preferences, activities and relationships are likely to change. Additionally, young adults are most likely to drink socially in public places (Hardie et al., 2023), making them significant and easily accessible targets for public health efforts. Consequently, it is important to continue to track trends in adolescent drinking into young adulthood to understand whether and why the decline in drinking within this generation persists.

Should the decline in adolescent drinking continue into young adulthood and beyond, it would be expected that alcohol-related harms would reduce significantly in the future (Aiken et al., 2018; Livingston & Vashishtha, 2019). Some researchers also suggest that even a shift towards later initiation of alcohol consumption may reduce alcohol-related harms in later life, though evidence for this is weak due to a paucity of studies able to make strong causal claims regarding the long-term effects of early initiation and the attenuation of any effects after adjustment for confounding factors within the existing studies (Enstad et al., 2019; Kim et al., 2017; Maimaris & McCambridge, 2014).

The decline in adolescent drinking, initially noted in the US, has been documented since the early 2000s (Pape et al., 2018; Vashishtha et al., 2021), thus the earliest cohorts who drove the decline have now progressed through young adult-hood (considered here to be 18–25 years). While there are several studies that have examined trends in adolescent drinking and provided explanations to declining trends among adolescents, there has not been equivalent research amongst the young adult population (Kraus et al., 2018; Vashishtha et al., 2021). Previously, Livingston and Vashishtha (2019) examined consumption trends in young adults and found mostly mixed evidence for a decline in alcohol consumption among young adults. However, this research was not entirely systematic, comparing different age groups and consumption measures, and was based solely on literature which was readily accessible in the public domain (Livingston & Vashishtha, 2019). There has not yet been an attempt to systematically review the literature on trends in young adult drinking.

This is the first systematic review to comprehensively examine the trends in the prevalence of young adult alcohol consumption over time. The primary aim of the review was to determine whether the proportion of young adults aged 18–25 years who consume alcohol has changed over time since 2000 in selected high-income countries which have seen a decline in adolescent drinking. Prevalence of alcohol consumption was chosen as the outcome of interest as it has fallen substantially among adolescents, is commonly measured, and is related to overall population risk (Rossow & Romelsjö, 2006; Sobell & Sobell, 2004). Whether there was a difference between males and females in how prevalence changed over time was considered as a secondary outcome.

## Materials and methods

This review was conducted in accordance with the Preferred Reporting Items for Systematic Reviews and Meta-Analyses (PRISMA) guideline (Page et al., 2021). The protocol for the review is available in Supplementary Material 1 and was not pre-registered.

### Eligibility criteria

As this review involves prevalence data, we used the condition-context-population (CoCoPop) framework to focus the research question (Munn et al., 2015). Full eligibility criteria are shown in Table 1.

### Condition (variable of interest)

Studies were eligible if they reported the total, male or female prevalence of alcohol consumption or abstention in a country of interest. This must have been reported over a given time period (e.g. past week). Figures reported in papers as abstention were converted to consumption by calculating (100%-proportion of abstainers). Initial plans were to include heavy/binge drinking measures however resource constraints meant that this was not possible after searches were completed (see protocol changes in Supplementary Material 1).

### Context

The time period of interest was 2000 or later to ensure data was captured from the period after the initial adolescent drinking decline had been noted (Pape et al., 2018; Vashishtha et al., 2021). The included surveys needed to report 2 data points over a minimum 3-year time span to ensure some potential for medium-term change. Surveys with multiple cross-sectional reports across the study period were eligible for inclusion.

The countries of interest were English-speaking, high-income countries which have seen a decline in adolescent drinking (Kraus et al., 2018; Oldham et al., 2018; Pape et al., 2018; Vashishtha et al., 2021). High-income countries were defined in line with the World Bank categorization (The World Bank, n.d.). English-speaking countries were chosen for two key reasons. Firstly, many of the countries which have seen some of the earliest and steepest declines in adolescent drinking are English-speaking nations (Vashishtha et al., 2021). Choosing countries which have had early, steep declines in adolescent drinking ensures a longer time period to assess any concomitant changes in young adult drinking and a greater likelihood of such changes occurring. Additionally, scoping searches identified that a high proportion of records would come from grey literature, which is usually published in a country’s national language. English-speaking countries were chosen as time and resource constraints precluded translation of records from other languages into English.

Given these geographical and technical constraints, records from a total of six countries were eligible for inclusion: Australia, Canada, Ireland, New Zealand, the United Kingdom (UK), and the United States (US). The United Kingdom has four constituent nations: England, Northern Ireland, Scotland, and Wales (UK Parliament, 2016). Each country has its own surveys with relatively few UK-wide surveys on alcohol consumption. Consequently, trends in these constituent nations were considered separately where appropriate.

### Population

There was no clear *a priori* definition of ‘young adulthood’; the core age range of 18–25 was chosen as this has precedent in other related literature (Drazdowski, 2016; Kinouani et al., 2020; Petker et al., 2019; Scott et al., 2018). For reports to be eligible, all recorded ages within the survey or the particular survey age group band had to be between 16 and 34, including at least one year in the core 18–25 range. For example, an age group of 25–34 years would be included as it included one year (25 years) in the core range, whereas an age group of 26–34 years would not have been included. The exception to this was where the highest age within the sample was 18 years (for example, 16–18-year-olds); these results were not included as they were felt to reflect adolescent, rather than young adult, age groups.

General population and higher education (college) student populations, which have a separate acknowledged drinking culture but comprise a significant proportion of the available estimates in some countries (National Institute on Alcohol Abuse and Alcoholism, 2014), were included in this review. College populations were included only where results were available from national or regional studies rather than single institutions, to ensure that the population was more representative of the college population of the country. Other narrowly defined populations, such as those with a long-standing medical condition, were excluded.

### Search strategy

Electronic database searching was conducted in Medline, PsycInfo, and CINAHL from January 2000 to April 2021, using terms and synonyms for four concepts: alcohol consumption, young adults, trends over time, and the countries of interest. Search terms were combined to identify records which contained at least one term from each concept and additional search terms were used to exclude commonly identified irrelevant topics. Search strategies can be found in Supplementary Material 2.

Where it appeared that prevalence data had been collected as part of the study process but was not reported in the record, we first determined whether that information was available from another source such as a survey report. On the single occasion where information was not available from an alternative source, the corresponding author was contacted via email. When multiple articles used the same survey source, the author of the most recent report was contacted. Where no reply was received or data was not available, this record was excluded from the review.

Full reference list screening was conducted for included records and forward searches of the first 50 citations for each article on Google Scholar were completed to determine if any further articles were eligible for inclusion. A list of potential grey literature sources was compiled using an initial scoping search of ‘[country] +young adult + alcohol + survey’ on Google. Additional sources were identified through informal discussion with international researchers and other stakeholders within the authors’ network. A final version of this list is available in Supplementary Material 3.

### Screening and data extraction

One reviewer conducted most of the screening and data extraction (JD). A second reviewer (KR) independently assessed 15 randomly selected papers from the three database searches against the documented eligibility criteria at the ‘title and abstract’ stage, with full agreement between the two reviewers. Data were extracted using a standardized form which was developed *a priori*. Data extracted included record identifying information, funding sources and conflict of interest, country, study design, population, survey used, sample size, time measure and prevalence of alcohol consumption and/or abstention. Where data were missing or unclear, no attempts were made to contact authors due to the time constraints of this review. Instead, this missing or unclear data is commented upon throughout the document.

We used the Joanna Briggs Institute Checklist for Prevalence Studies (The Joanna Briggs Institute, 2017) to assess the risk of bias in included records. This carried the advantage of allowing the assessment of multiple types of study design with the same tool. The risk of bias for each record was assessed at the outcome-level by a single reviewer (JD). This tool includes nine domains. The overall quality of each outcome was assessed qualitatively rather than arriving at an overall score. Where multiple surveys were included in the same record, each survey was assessed separately. Similarly, where multiple waves of surveys were included, quality assessment was undertaken on each wave separately. When considering response rates, an overall survey response rate of 50% or above was generally considered to be sufficient. Some judgement was used with this figure, however, given that relatively low response rates can be justified if the sample remains representative of the population being studied (Bethlehem et al., 2009; Hendra & Hill, 2019; Wright, 2015). Coverage of the population, another domain on the checklist, was used as a marker of quality. It was assessed by comparing the proportion of survey respondents in the young adult age band(s) to the estimated proportion of the population who fall into those age bands. Young adults tend to be under-represented in general population surveys, therefore a coverage rate of approximately 5% below the proportion of that age group in the general population was considered acceptable.

### Synthesis of results

Meta-analysis was precluded at the scoping stage by the considerable heterogeneity in survey methods, population, age groups and time measure for the primary outcome, which was assessed qualitatively. As such, results are presented narratively at the survey-level, being divided into five population groups following data extraction: 18–25 years (US), 18–25 years (other countries), 18–35, 16–24 and college (higher level educational institutions). We chose these groupings based on a mix of frequency of use in survey reports and to separate out the US from other countries in the 18–25 group given the higher legal purchase age of 21 in the US. The results focus on the surveys with a minimum 10-year time span to ensure sufficient and robust trend data are available. The remaining surveys with time spans between 3- and 10-years are presented in Supplementary Material 4 and are discussed briefly throughout.

The primary outcome is presented as a change in the prevalence of alcohol consumption (% of total survey population) between the survey start year (first cross-sectional study available from 2000 onwards) and end year (last cross-sectional study available). To avoid overstating small changes, we chose to describe changes in prevalence of >5% as increases or decreases over time and changes of ≤5% as a stable prevalence over time. A secondary outcome comparing the difference in changes in alcohol consumption between sexes (male and female) was also considered.

Any reporting on the statistical significance of trends is reported at record-level (Supplementary Material 5). Most records use a *p*-value of ≤.05 for statistical significance, with the exception of White et al. (2015) who use a cut off of ≤0.01. The commentary also includes consideration of whether participants were of the legal minimum purchase age (MPA) for alcohol for the countries included (Table 2). Whilst this is not the same as the minimum legal age for alcohol consumption, many countries do not have an age limit on consumption *per se*, particularly outside licensed premises.

## Results

### Description of included studies

**Figure 1:**
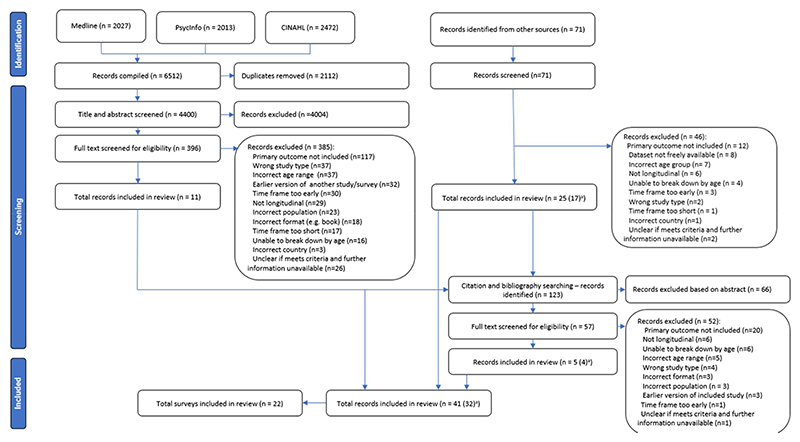
PRISMA (Preferred Reporting Items for Systematic Reviews and Meta-Analyses) flow diagram. ^a^Seven surveys included data from two or more reports on a single wave or year (Australian Bureau of Statistics, 2002, 2017–18; Bromley et al., 2008; Central Statistics Office, 2016, 2020; Central Survey Unit, 2006, 2008; Government of Canada, 2016, 2021; Ministry of Health, n.d.; Information Analysis Directorate, 2014; Schoenborn et al., 2004, 2013; Scottish Health Survey, 2020; The Scottish Government, 2009). We treated these reports as a single record for synthesis purposes but as separate records for quality assessment.

In total, 4400 records were identified from initial database searches conducted in April 2021, as can be seen in the PRISMA diagram (Figure 1). Additional grey literature and bibliography/citation searches identified a further 194 records, bringing the total to 4594 records identified. Of these, 41 records were included in the review. The main reasons for screening out records were a lack of inclusion of the primary outcome or failing to meet eligibility criteria regarding age categories, population, or required time periods (Table 3).

We included data from 22 surveys. For seven of these, we included data from two or more reports on a single wave or year. We treated these reports as a single record for synthesis purposes but as separate records for quality assessment. The 22 surveys therefore provided 32 records for synthesis and 41 for quality assessment. Record-level data is available in Supplementary Material 5 and further information about each survey – including the population, time period, measure, survey mode, and response rates – is available in Supplementary Material 6.

Of the 32 records used for synthesis, 31 included general population data. Two of these also included a subset of data from the 18–22-year-old US college population. The remaining record reported on the US college population only. Twenty-three (72%) records reported on alcohol consumption, mostly over the past year (*n* = 13, 41%), or the past month (*n* = 8, 20%). Fourteen (44%) records reported alcohol abstention figures, mostly over the past-year (*n* = 7, 22%) or lifetime abstention (*n* = 6, 19%). Forty-one percent of records (*n* = 13) were from the US. The remaining records included eight records (25%) from the UK, five records (16%) from Australia, two records (6%) from New Zealand, two records (6%) from Ireland and two records (6%) from Canada.

The two Canadian surveys included are closely related as, although there are some methodological differences, the key questions regarding the prevalence of alcohol consumption remain consistent over time (Canadian Centre on Substance Use and Addiction, 2017; Student Drug Use Surveys Working Group, 2013). This allowed for some tentative comparisons between the surveys, in line with the Canadian government reporting of this data (Government of Canada, 2020). Therefore, comparisons across both surveys are discussed in the text where relevant, with the results of both surveys reported in Supplementary Material 4 (as both surveys are under 10 years in length).

Study time periods ranged from 3 to 19 years. Ten records had relatively short durations of under 10 years. The results of these shorter duration surveys can be found in Supplementary Material 4. The results of the remaining 22 records are discussed below.

### Risk of bias assessment

The quality of included records varied by domain of the checklist (Supplementary Material 7). All records used appropriate sampling frames and techniques, had an adequate sample size, and were assessed as reliably conducted using valid methods. Many studies, however, had important limitations. Firstly, many records did not adequately describe the population of interest in sufficient detail (29/41, 71%), considered here to be a sex breakdown for the age group in question. This made it difficult to determine how comparable populations were to each other, either between different surveys or for the same survey at different time points. Additionally, as is explored below, some surveys found differing trends for males and females. The relative proportions of males and females within the age group may therefore have impacted on the total population trend. While we can generally assume that there is likely to be an approximately equal proportion of males and females (50:50) in a well-sampled general population survey, where this was not reported we could confirm this.

Most records (31/41, 76%) reported adequate overall response rates, with the remainder not having response rates available in the public domain. The majority (31/41, 76%), however, did not include response rate or coverage by age, increasing uncertainty regarding whether the sample were representative of the young adult age group. Secondly, 80% of records (33/41) did not clearly report the numerator and denominator for prevalence data. This made it difficult to check the data, determine an accurate annual young adult sample size for whole population surveys and to calculate the non-response rate to alcohol-specific survey questions.

### Trends in drinking prevalence

#### 18–25 Years

**Figure 2:**
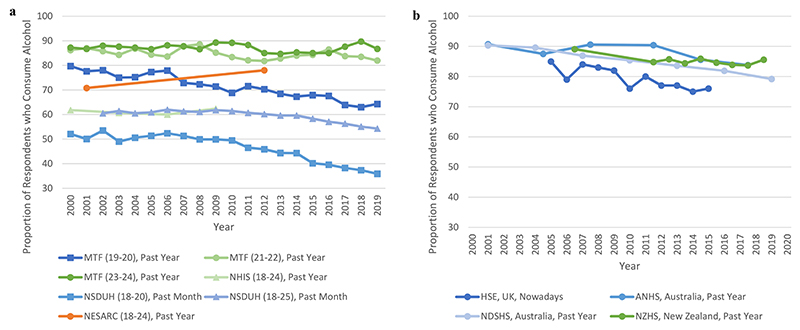
(a) The prevalence of alcohol consumption amongst 18–25-year-olds in the United States by consumption measure, 2000–2019 (Chen & Yoon, 2021; Dawson et al., 2015; Hasin et al., 2019; White et al., 2015; Schoenborn et al., 2004, 2013; Schulenberg et al., 2020; Slater et al., 2015; Substance Abuse and Mental Health Services Administration, 2020). (b) The prevalence of alcohol consumption amongst 18–25-year-olds in Australia, New Zealand and the United Kingdom, by country and consumption measure, 2000–2019/20 (Australian Bureau of Statistics, 2002, 2017–18; Australian Institute of Health and Welfare, 2021; Livingston, 2015; Ministry of Health, n.d.; Ng Fat et al., 2018). Orange lines represent an increase in prevalence of consumption of > 5% over the survey time frame; blue lines represent a decrease in prevalence of consumption of > 5% over the survey time frame; and green lines represent a stable prevalence of consumption of (change of ≤5%) over the survey time frame. (a) MTF: Monitoring the Future; NESARC: National Epidemiological Survey on Alcohol and Related Conditions; NHIS: National Health Interview Survey; NSDUH: National Survey on Drug Use and Health. (b): ANHS: Australian National Health Survey; HSE: Health Survey for England; NDSHS: National Drug Strategy Household Survey; NZHS: New Zealand Health Survey.

Thirteen surveys reported on prevalence of alcohol consumption amongst 18–25-year-olds. Of the four American surveys (Table 4), the two which reported on 18–20-year-olds (under MPA in the US) found a decrease in total prevalence of consumption over time. For example, the prevalence of past-month consumption amongst 19–20-year-olds in one survey fell from 59.1% to 45.6% between 2000 and 2019 (Schulenberg et al., 2020). By contrast, results for US surveys where some or all adults were of MPA were more mixed with increases, decreases, and stability noted across different time measurements (Figure 2(a)). When considering males and females, most survey results indicate the same direction of trends for both sexes, except in the Monitoring the Future (MtF) results which indicated a decrease in prevalence for males but stability for females (Schulenberg et al., 2020).

When considering other countries (Table 5 and Figure 2(b)), survey results from the UK and Australia with time spans over 10 years indicated a mostly downward trend in total prevalence of alcohol consumption. The exception to this was the lifetime prevalence trends in the Australian National Health Survey (ANHS), which indicated stability (Australian Bureau of Statistics, 2002, 2017–18). These stable results were also mirrored in the New Zealand Health Survey’s past year prevalence trends (Ministry of Health, n.d.).

For survey results from studies with a less than 10-year timespan in this age group, the findings were also mostly stable. The only exceptions were an increase in female ‘nowadays’ consumption in the UK and a decrease in males past-year consumption in New Zealand.

#### 18–35 Years

The seven US surveys that reported on older age groups showed mixed results (Table 6). Four surveys found no change, or mostly no change, in the prevalence of consumption over time. Two surveys found an increase in consumption over time. The final survey, Monitoring the Future, had stable results for lifetime and past year measures, but increased prevalence for overall past month and female past month measures. Two survey results show an increase in the prevalence of female consumption on a background of stable male consumption. For example, between 2006 and 2018, the prevalence of past-year consumption amongst 18–29-year-old females rose by 9% points from 61% to 70%, whilst the prevalence of male past-year consumption remained stable (McKetta & Keyes, 2019).

The six surveys from the UK, Ireland, Australia, and New Zealand generally found declines or stability in total prevalence of young adult alcohol consumption (Table 6). In contrast to data from the US, when male and female consumption patterns differed, female consumption generally followed a downward trend while male consumption remained stable. This was most notable in the ANHS where the prevalence of female past-year consumption fell by 11.4% points between 2001 and 2017/8, while the prevalence of male consumption fell just 1.7% points, remaining stable (Australian Bureau of Statistics, 2002, 2017–18). Examination of the full trend for all data points (data available on request), however, found that this increasing difference in prevalence between sexes is only consistent in the ANHS.

Surveys in New Zealand, Ireland, and some in the UK (England and Wales) with a time span under 10 years generally reported stability in the prevalence of consumption. The Adult Drinking Patterns in Northern Ireland survey noted a decrease in past week measures, and stability for males but a decrease for females in past year prevalence of consumption (Central Survey Unit, 2006, 2008; Information Analysis Directorate, 2014).

#### 16–24 Years

Three surveys from the UK reported on 16–24-year-olds with a time span over 10 years, most of which found a decrease in prevalence of alcohol consumption over time, with two ‘nowadays’ measures resulting in stable findings (Table 7). Generally, the decline in prevalence of consumption was relatively similar between males and females, with the notable exception of the Opinion and Lifestyle Survey. Here, the prevalence of past week consumption amongst British males fell by 16% points from 64% to 48% between 2005 and 2017, double the 8% point decrease seen amongst females and resulting in equal prevalence between sexes at survey end point (Office for National Statistics, 2018). Both surveys from the UK with a time span under 10 years also found decreases in prevalence of alcohol consumption over time.

#### College (18–22 years)

Two US surveys reported on the college population, both of which found a decrease in the prevalence of alcohol consumption over time (Table 8). For example, NSDUH found an 8 percentage point fall in the prevalence of past-year consumption from 80.0% to 72.0% between 2002 and 2018 (McCabe et al., 2021).

## Discussion

### Principal findings

This is the first systematic review to comprehensively examine trends in the prevalence of young adult alcohol consumption over time. Outside of the US, studies generally showed clear declines (such as in Australia and Ireland), or a mix of stable and declining trends (in the UK), while New Zealand showed stable trends. Steep declines were especially evident within England compared to the rest of the UK, and in the 18–25 population within Australia. Studies under a 10-year time span generally showed stable trends across all countries.

Evidence from North America was more mixed, finding a decrease in prevalence of consumption among adults below MPA and among college-specific populations, but mixed trends for adults above the MPA. Of the seven surveys based in the US for adults above the MPA, three reported stable findings, two reported increases in prevalence, and two reported mixed findings based on survey measures and age ranges.

Some surveys, mainly from the US, found a convergence in the prevalence of consumption between sexes, but findings on this were not consistent. The included surveys were, however, heterogenous and the quality of many was limited by a lack of clear reporting on the size, response rate, and/or representativeness of the age-specific population.

We found that trends in young adult drinking in Great Britain, Ireland, and Australia generally mirrored adolescent drinking trends, which appears to suggest a sustained decline in alcohol consumption into young adulthood. Studies in Australia and Sweden have explicitly examined cohort consumption patterns and shown that declines in adolescent drinking continue into young adulthood but that cohort differences shrink, suggesting partial ‘catching up’ (Kraus et al., 2023; Livingston et al., 2021). If high-quality longitudinal surveys confirm this finding, this may have potential benefits going forward for both health and wider societal well-being (Oldham et al., 2018; WHO, 2018). It is important to note, however, that alcohol consumption remained high, with the majority of young adults of MPA in all surveys reporting consumption of alcohol within the past month.

The variable trends seen in the US are more difficult to interpret, as are the reasons why these trends diverged from the pattern seen in other countries included in this review. To some extent, this may be related to methodological differences between surveys. Additionally, demographic, political and cultural differences between and within countries could be important (Beard et al., 2017; Livingston, 2015; Pennay et al., 2019; Sudhinaraset et al., 2016). Taken with the decline in adolescent consumption, any increase in consumption amongst young adults may represent a shift towards later attainment of milestones, including initiation of alcohol consumption, as part of a ‘delayed adulthood’ (Hayford & Furstenberg, 2008; Twenge & Park, 2019).

Furthermore, the US has historically had lower levels of alcohol consumption, particularly among women, than the other countries in this review. Therefore, longer-term shifts towards increased alcohol consumption may be overlapping with the more recent shifts in youth drinking to produce trends that are distinct to the US. This divergence in trends between the US and other included countries appeared to result in a convergence towards a more similar prevalence of consumption between countries. This may actually suggest a homogenization of drinking cultures between countries, which has already been noted to some extent across the European Union (Bentzen et al., 2001; Smith & Mitry, 2007; Smith & Solgaard, 2000).

The differences in alcohol consumption by sex are also interesting. The more mixed picture in the US appeared to be driven by stable male consumption and increasing female consumption. Reasons for this are unclear. Again, the methodological weaknesses within the surveys may have a role. Additionally, gendered societal norms, both around drinking culture and wider social roles could have played a part (Keyes et al., 2008, 2019; Slade et al., 2016). The longer-term consequences of this converging trend between sexes could have potential practice-related implications with a need to raise awareness amongst health and social care practitioners of this changing demographic trend, along with careful monitoring of consumption and harm trends to inform future service planning.

### Strengths and limitations

This is the first review to systematically synthesize findings on trends in the prevalence of young adult alcohol consumption and abstention. Literature searches identified over 4000 records, with over 30 included in the review. Records included data from large national surveys with rigorous sampling methodologies, so the trends identified are likely to be representative for the target population within each country. The lack of reported annual sample sizes and response rates for the age-specific survey populations do, however, make it difficult to determine whether certain demographic groups were under-represented (Meiklejohn et al., 2012; Nolen-Hoeksema, 2004).

Furthermore, the considerable methodological heterogeneity between surveys makes it difficult to directly compare results (Boniface & Shelton, 2013; Nugawela et al., 2016). The introduction of a large multinational study examining trends in alcohol consumption, similar to the adolescent European School Survey Project on Alcohol and Other Drugs (ESPAD), could help to address some of the methodological heterogeneity between surveys and may also allow comparison with additional non-English speaking countries which were excluded from this review, but would have significant logistical challenges (ESPAD Group, 2020).

Also, we chose to compare only data from the start and end year of each survey. There is also a risk that the data points used may not accurately represent the overall trend, however, since the data sources used were primarily large national surveys, the risk of high point-to-point variability is low.

Additionally, the self-reported nature of surveys increases the risk of social desirability and non-response biases, which could over- or under-estimate prevalence. There is evidence to support high non-response rates in both frequent drinkers and abstainers, so the impact of non-response on both individual survey responses and changes over time is difficult to assess (Boniface & Shelton, 2013; McCabe & West, 2016; Nugawela et al., 2016).

Also, this review focuses solely on one aspect of alcohol consumption: overall prevalence. Overall prevalence was chosen as it has fallen substantially among adolescents, is commonly measured, and is related to overall population risk. Further exploration and synthesis of literature on wider drinking habits would provide additional insights particularly with regard to the long-term health implications of these trends, especially as some studies point to differences in trends for heavy episodic drinking compared to any drinking (Kraus et al., 2023).

Finally, none of the included studies contained data from 2020 onwards. The COVID-19 pandemic has already had a short-term effect on drinking patterns in the young adult population. It would be valuable to examine the impact the pandemic has had on longer-term trends in consumption, although these are unlikely to be understood for several years.

### Conclusions

Overall, we found evidence that young adult drinking is generally declining in countries outside of the US (i.e. UK, Ireland, Australia), but that trends are more mixed in North America. These findings suggest that previously identified declines in adolescent drinking are being maintained into young adult-hood and should lead to reduced rates of alcohol-related health and social harms in the future. There remain some key gaps in the evidence (e.g. for young adults in the UK) and reporting practices vary substantially between jurisdictions and surveys. High-quality, multinational surveys for these populations, similar to those conducted for adolescents (ESPAD Group, 2020; WHO, 2018) or based on the Standard EU Alcohol Survey (DEEP SEAS, n.d.), may help to improve the quality of this evidence.

## Supplementary Material

Supplementary material

## Figures and Tables

**Table 1:** Condition-Context-Population framework.

Element	Description	Key Concept for this Systematic Review	Inclusion Criteria	Exclusion Criteria
**Co**ndition	Variable of interest	Proportion of the population who consume alcohol. (Search terms were also used to search for the inverse – proportion of the population who do not consume alcohol)	Includes primary outcome (% of the population who do (not) consume alcohol)	–
**Co**ntext	Context or specific setting such as geographical and time parameters	*Geography:* English-speaking high-income countries in Europe, North America and Australasia which have seen a decline in adolescent drinking * Europe:* Ireland and UK * North America:* Canada and US * Australasia:* Australia and New Zealand *Time:* Minimum 3-year time span, with the two data points from 2000 or later	Includes national data from a country or countries of interest *Europe:* Ireland and UK (England, Northern Ireland, Scotland or Wales) *North America:* Canada and US *Australasia:* Australia and New Zealand Minimum 3-year time span, with the two data points from 2000 or later	Subnational data (e.g. individual state-level survey)
**Population**	Appropriate population	Young adults (core age 18–25 years, range 16–34 years) from a general or student population	General population or school/university student population Age range: must include an age in the range 18–25 years (minimum age in group used for data extraction 16 years, maximum age 34 years)	Not general or student population – examples include populations comprised of individuals with specific medical conditions
Other	–	–	Repeated cross-sectional survey Journal article, dissertation, survey report or online survey data from the survey’s author (for example, using a data explorer) English language	Cohort survey Any other type of information source such as book chapters and other systematic reviews

**Table 2:** Minimum age (years) to purchase alcohol by country (International Alliance for Responsible Drinking, 2022).

Country	Minimum Purchase Age (Years) for Alcohol
Australia	18
Canada – Alberta, Manitoba, and Quebec	18
Canada – British Columbia, Newfoundland and Labrador, New Brunswick, Nova Scotia, Northwest Territories, Nunavut, Ontario, Prince Edward Island, Saskatchewan and Yukon	19
Ireland	18
New Zealand	18 (reduced from 20 in December 1999, Gruenewald et al., 2015)
United Kingdom	18
United States	21 (for all states since 1984, APIS, n.d.)

**Table 3:** Characteristics for each record and survey.

Study characteristic		Number of included records (% of all 32 included records)^a,b^	Number of included surveys (% of all 22 included surveys)^a^
Age Group	18–25	19 (59)	13 (59)
	16–24	5 (15)	5 (23)
	18–35	18 (56)	17 (77)
	College (18–22)	3 (9)	2 (9)
Measure	Consumption	23 (72)	18 (82)
	Past-year	13 (41)	14 (64)
	Past-month	8 (25)	4 (18)
	Past-week	4 (13)	4 (18)
	Lifetime	2 (6)	2 (9)
	“Nowadays”	2 (6)	1 (5)
	Abstention	14 (44)	8 (36)
	Past-year	7 (22)	6 (27)
	Past-week	2 (6)	1 (5)
	Lifetime	6 (19)	5 (23)
	“Nowadays”	2 (9)	2 (9)
Country	United States	13 (41)	7 (32)
	United Kingdom	8 (25)	7 (32)
	Australia	5 (16)	2 (9)
	New Zealand	2 (6)	2 (9)
	Ireland	2 (6)	2 (9)
	Canada	2 (6)	2 (9)
Duration of Change Period	3–5 years	6 (19)	
	6–10 years	8 (25)	
	11–15 years	12 (38)	
	16–20 years	10 (32)	
Source	Journal article	11 (34)	
	Data sheet/explorer	11 (34)	
	Report	10 (32)	

a Numbers may not sum due to rounding or because records/surveys include multiple populations, time periods and/or measures.

b Seven surveys included data from two or more reports on a single wave or year (Australian Bureau of Statistics, 2002, 2017-18; Bromley et al., 2008; Central Statistics Office, 2016, 2020; Central Survey Unit, 2006, 2008; Government of Canada, 2016, 2021; Ministry of Health, n.d.; Information Analysis Directorate, 2014; Schoenborn et al., 2004, 2013; Scottish Health Survey, 2020; The Scottish Government, 2009). We treated these reports as a single record for synthesis purposes but as separate records for quality assessment. Data from the more recent record considered here for ‘source’ purposes.

**Table 4:** Prevalence of consumption amongst US 18–25 year olds.

US 18-25								
				18–20				
Survey		Age Range (Years)		Years	Measure	Prevalence (%)	% Change	Trend
Monitoring the Future (Schulenberg et al. 2020)		19–20a		2000–2019	Past month	T: 59.1-45.6	−13.5	↘
					Past year	T:79.7-64.3	−15.4	
National Survey on Drug Use and Health		18–20		2000–2019 (Chen & Yoon, 2021; Hasin et al., 2019; Slater et al., 2015; White et al., 2015)	Past month	T: 49.0-35.9	−13.1	↘
						M: 52.4–33.8	−18.6	
						F: 45.4–38.2	−7.2	
				2002–2013	Lifetime^b^	M: 82.4–74.2	−8.2	
				(White et al., 2015)		F: 81.0–74.6	−6.4	
				**21-25**				
Monitoring the Future (Schulenberg et al., 2020)		21–22		2000–2019	Past month	T: 70.5–68.4	−2.1	↔
					Past year	T: 86.2–82.0	−4.2	
		23–24			Past month	T: 71.5–73.8	+2.3	
					Past year	T: 87.2–86.7	−0.5	
National Survey on Drug Use and Health (White et al., 2015)		21–25		2002–2012	Past month	M: 72.6–72.8	+0.2	↔
						F: 60.6–65.4	+4.8	
					Lifetime^b^	M: 91.6–90.9	−0.7	
						F: 88.4–90.1	+1.7	
				**18-25**				
Monitoring the Future (Schulenberg et al., 2020)		19–22		2000–2019	Past month	M: 67.8–56.4	−11.4	M: ↘
						F: 62.2–58.2	−4.0	F: ↔
National Epidemiological Survey on Alcohol and Related Conditions (Dawson et al., 2015)		18–24		2001/2–2012/13	Past year	T: 70.8–78.0	+7.7	↗
National Health Interview Survey (Schoenborn et al., 2004, 2013)		18–24		1999–2001–2008–2010	Past year	T: 61.8–62.4	+0.6	↔
						M: 67.1–65.9	−1.2	
						F: 56.6–58.9	+2.3	
					Lifetime	T: 66.5–66.6	+0.1	↔
						M: 70.7–69.5	−1.2	
						F: 62.4–63.8	+1.3	
National Survey on Drug Use and Health		18–25		2002–2019 (SAMHSA, 2020)	Past month	T: 60.5–54.3	−6.2	↘
				2002–2012 (White et al., 2015)	Past month	M: 65.2–62.6	−2.6	↔
						F: 55.7–57.9	+2.2	
					Lifetime^b^	M: 87.8–84.4	−3.4	↔
						F: 85.5–84.4	−1.1	

Key: ↗: Increase of >5.0% between the start and end point provided. ↘: Decrease of >5.0% between the start and end point provided. ↔: Stable – any value with an increase or decrease of ≤5% between the start and end point provided. T: total; M: male; F: female.

a Monitoring the Future consists of a cross-sectional survey in 12^th^ grade, with participants then selected to continue in a “panel” survey. This therefore has both cross-sectional and cohort elements.

b Reported in record as abstention data.

**Table 5:** Prevalence of consumption amongst 18–25 year old from Canada, United Kingdom, Australia and New Zealand.

Other Countries 18-25							
Country	Survey	Age Range (Years)	Years	Measure	Prevalence (%)	% Change	Trend
UK (England)	Health Survey for England	18–24	2005–2015	“Nowadays”^a^	T: 85–76	−9.0	↘
	(Ng Fat et al., 2018)						
Australia	Australian National Health Survey (Australian Bureau of Statistics, 2002), 2017–18)	18–24	2001–2017/8	Past week^b^	T: 63.3–48.4	−14.9	↘
					M: 68.3–52.6	−15.4	
					F: 58.1–44.0	−14.1	
				Past year^b^	T: 90.7–83.8	−6.9	↘
					M: 91.6–84.4	−7.2	
					F: 90.0–83.3	−6.7	
				Lifetime^b^	T: 93.3–88.6	−4.7	↔
					M: 93.5–89.4	−4.1	
					F: 93.0–88.0	−5.0	
	National Drug Strategy Household Survey	18–24	2001–2019 (AIHW, 2021)	Past year	T: 90.3–79.2	−11.1	↘
			2001–2013 (Livingston, 2015)	Lifetime^a^	T: 92.5–86.3	−6.2	
New Zealand	New Zealand Health Survey	18–24	2006/7–2018/19 (Ministry of Health, n.d.)	Past year	T: 89.1–85.6	−3.7	↔

Key: ↗: increase of >5.0% between the start and end point provided; ↘: Decrease of >5.0% between the start and end point provided; ↔: Stable – any value with an increase or decrease of ≤5% between the start and end point provided. UK: United Kingdom; T: total; M: male; F: female.

a Author-calculated value based on data available in the original record.

b Reported in record as abstention data.

**Table 6:** Prevalence of consumption amongst 18–35-year-olds.

18–35 Years							
Country	Survey	Age Range (Years)	Years	Measure	Prevalence (%)	% Change	Trend
US	Behavioral Risk Factor Surveillance System (Grucza et al., 2018)	18–29	2002–2013	Past month	T: 60.5–56.1	−4.4	↔
	Monitoring the Futurea	19–30	2000–2015 (Twenge & Park, 2019)	Lifetime^b^	T: 89.5–86	−3.5	↔
		23–26	2000–2019 (Schulenberg et al., 2020)	Past month	M: 76.2–74.9	−1.3	M: ↔
					F: 65.8–74.1	+8.3	F: ↗
		25–26		Past month	T: 68.7–75.0	+6.3	↗
				Past year	T: 84.2–87.8	+3.6	↔
	National Alcohol Survey (Grucza et al., 2018)	18–29	2000–2010^c^	Past year^d^	T: 69.3–75.3	+6.0	↗
	National Epidemiological Survey on Alcohol and Related Conditions (Grant et al., 2017; Grucza et al., 2018)	18–29	2001/2–2012/3	Past month	T: 73.1–80.1	+7.0	↗
	National Health and Nutrition Examination Survey (Grucza et al., 2018)	18–29	2001/2–2013/4	Past year^d^	T: 75.7–80.1	+4.4	↔
	National Health Interview Survey	18–29	2002–2013 (Grucza et al., 2018)	Past year	T: 65.1^d^–68.3^d^	+3.2	↔
			2006–2018 (McKetta & Keyes, 2019)		M: 73–72	−1.0	M: ↔
					F: 61–70	+9.0	F: ↗
	National Survey on Drug Use and Health	18–29 (Grucza et al., 2018)	2002–2012	Past year	T: 78.2–78.7	+0.5	↔
				
				
UK (England)	Health Survey for England (NHS Digital, 2020)	25–34	2000–2019	Past week^e^	T: 72–51	−21.0	↘
					M: 77–57	−20.0	
					F: 67–45	−21.0	
UK (Scotland)	Scottish Health Survey**	25–34	2008–2019	Past year	T: 90–88	−2.0	T: ↔
			(Scottish Health Survey, 2020; The Scottish Government, 2009)				M: ↔
							F: ↘
			2003-2019 (Bromley et al., 2008; Scottish Health Survey, 2020)		M: 93 to 92	−1.0	
					F: 90 to 84	−6.0	
Ireland	Irish National Drug and Alcohol Survey (Mongan et al., 2021)	25–34	2002/3–2019/20	Past year	T: 91.0–81.3	−9.7	↘
Australia	Australian National Health Survey (Australian Bureau of Statistics, 2002, 2017–18)	25–34	2001–2017/18	Past week^f^	T: 63.2–52.1	−11.1	↘
					M: 73.0–61.3	−11.7	
					F: 53.7–43.5	−10.2	
				Past year^f^	T: 87.3–80.4	−6.9	T: ↘
					M: 90.0–88.3	−1.7	M: ↔
					F: 84.7–73.3	−11.4	F: N
				Lifetime^e^^,^^f^	T: 92.5–88.4	−4.1	T: ↔
					M: 93.9–92.6	−1.3	M: ↔
					F: 91.2–83.8	−7.4	F: ↘
	National Drug Strategy Household Survey	18–29	2001–2013 (Callinan et al., 2017)	Lifetime	T: 87.0–79.0	−8.0	↘
		20–29	2001–2019	Past year	T: 91.1–78.0 (AIHW, 2020)	−12.1	
		25–29			T: 91.2–76.4 (AIHW, 2021)	−14.8	
			2001–2013 (Livingston, 2015)	Lifetime	T: 95.9–89.2	−6.7	
New Zealand	New Zealand Health Survey	25–34	2006/7–2018/19 (Ministry of Health, n.d.)	Past year	T: 86.7–82.3	−4.8	↔

Key: ↗: Increase of >5.0% between the start and end point provided. ↘: Decrease of >5.0% between the start and end point provided. ↔: Stable – any value with an increase or decrease of ≤5% between the start and end point provided. US: United States; UK: United Kingdom; T: total; M: male; F: female.

a Monitoring the Future consists of a cross-sectional survey in 12^th^ grade, with participants then selected to continue in a “panel” survey. This therefore has both cross-sectional and cohort elements.

b Figure obtained from a graph.

c Estimates are based on the fitted trend line for each survey, using all years of available data. These results were used to calculate the predicted average prevalence for each survey and each subgroup for the years 2002 and 2013.

d Note that the National Alcohol Survey takes place approximately every 5 years (2000, 2005, 2009/10, etc) and so did not have surveys in 2002 and 2013. The data here is based on a fitted trend line using all years of available data.

e Reported in record as abstention data.

f Author-calculated value based on data available in the original record.

**Table 7:** Prevalence of consumption amongst 16-24-year-olds.

16-24 Years							
Country	Survey	Age Range (Years)	Years	Measure	Prevalence (%)	% Point Change	Trend
UK (England)	Health Survey for England	16–24	2000–2019 (NHS Digital, 2020)	Past week^a^	T: 63–40	−23	↘
					M: 67–43	−24	
					F: 59–37	−22	
			2005–2015 (Ng Fat et al., 2018)	Lifetime^a^	T: 91–83	−8	
UK (Great Britain)	Opinion and Lifestyle Survey (Office for National Statistics, 2018)	16–24	2005–2017	Past week	T: 60–48	−12	↘
					M: 64-48	−16	
					F: 56–48	−8	
				“Nowadays”^a^	T: 81–77	−4	↔
					M: 83–78	−5	
					F: 80–76	−4	
UK (England)				Past week	T: 60–50	−10	↘
					M: 64–51	−13	
					F: 56–49	−7	
				“Nowadays”^a^	T: 81–77	−4	↔
					M: 83–78	−5	
					F: 79–76	−3	
UK (Scotland)	Scottish Health Survey	16–24	2008-2019 (Scottish Health Survey, 2020; The Scottish Government, 2009) 2003-2019	Past year^a^	T: 93–83	−10	↘
					M: 89–83	−6	
			(Bromley et al., 2008; Scottish Health Survey, 2020)		F: 90–83	−7	

Key: ↗: Increase of >5.0% between the start and end point provided; ↘: Decrease of >5.0% between the start and end point provided.↔: Stable – any value with an increase or decrease of ≤5% between the start and end point provided. UK: United Kingdom; T: total; M: male; F: female.

a Reported in record as abstention data.

**Table 8:** Prevalence of consumption amongst in the college population.

College Population							
Country	Age Range (Years)	Survey	Years	Measure	Prevalence (%)	Percentage Change	Trend
US	18–22 (1–4 years after high school)	Monitoring the Future^a^	2000–2019	Past month (Schulenberg et al., 2020)	T: 67.4–62.2	−5.2	↘
				Past year	T: 83.2–77.6	−5.6	
				(Schulenberg et al., 2020) Lifetime	T: 86.6–79.2	−7.4	
				(Twenge & Park, 2019)			
	18–22	National Survey on Drug Use and Health (McCabe et al., 2021)	2002–2018	Past year	T: 80.0^b^–72.0b	−8.0	↘

Key: ↗: Increase of >5.0% between the start and end point provided; ↘: Decrease of >5.0% between the start and end point provided; ↔: Stable – any value with an increase or decrease of ≤5% between the start and end point provided. UK: United Kingdom; T: total; M: male,; F: female.

a Monitoring the Future consists of a cross-sectional survey in 12^th^ grade, with participants then selected to continue in a “panel” survey. This therefore has both cross-sectional and cohort elements.

b Reported in record as abstention data.
