# Supplementary material for: Have declines in the prevalence of young adult drinking in English-speaking high-income countries followed declines in youth drinking? A systematic review
Source: Drugs (Abingdon Engl). 2024 May 22;32(1):15–28. doi: 10.1080/09687637.2024.2335989 (PMC7616612; doi:10.1080/09687637.2024.2335989)
Supplement: Supplemental Material [file IDEP_A_2335989_SM4808.pdf]

Supplementary Material

Supplementary Material 1 – Protocol

Has the proportion of young adults (aged 18-30 years) who consume alcohol changed over time in selected English-speaking high-income countries which have seen a decline in adolescent drinking?: a quantitative systematic review

Background

Globally, over a third of the world's population consume alcohol. Whilst much of this consumption occurs in moderation, significant harms can result from sustained alcohol use or episodes of high-intensity drinking. These harms can include short term effects such as injury as well as longer-term medical impacts including liver disease, certain cancers and depression. In addition, alcohol use and associated violence can have socioeconomic impacts on both the individual, their families and the wider community. The degree of harm that arises from alcohol use is significant, with alcohol use currently the leading cause of premature death in people aged 15-49 years old globally.

Recent trends from the United States, Canada, Australia and many European countries suggest that adolescents (usually ranging from 13 to 18 years old) are consuming less alcohol. There has been a decline in the proportion of the adolescent population who consume any alcohol; the rate of abstention amongst 14–17-year-olds in Australia, for example, increased from 32.9% to 50.2% between 2001 and 2010. Additionally, amongst alcohol drinkers, there has been a reduction how often adolescents are drinking and how many drinks they consume per session. The reasons for this decline are not yet clear. Increased use of social media, changing parenting styles and changes to alcohol policies are just some of the factors which may potentially play a role.

Should this decline in adolescent drinking continue into young adulthood and beyond, it would be expected that alcohol-related harms would also reduce significantly. This could have a huge impact to both the individuals concerned and society as a whole. The decline in adolescent drinking has been documented since the millennium shift, meaning that there should have been sufficient time to monitor trends into young adulthood. While there is a strong body of evidence about recent trends in adolescent drinking habits, however, there is less consistent data about what happens to alcohol consumption habits following the transition to young adulthood. The available evidence is sometimes contradictory, with research from New Zealand and the UK suggesting that the decline in adolescent drinking does continue into young adulthood, whereas evidence from the United States finding the reverse. There has not yet, to the best of the author's knowledge, been an attempt to systematically review the literature on trends in young adult drinking. This review attempts to fill the gap in the literature on this important topic.

Research Question

Question: Has the proportion of young adults (aged 18-30 years) who consume alcohol changed over time in selected high-income countries which have seen a decline in adolescent drinking?

Aim: To determine whether the proportion of young adults aged 18-30 years who consume alcohol has changed over time in selected high-income countries which have seen a decline in adolescent drinking

Objectives:

1. To systematically review the available literature, through database searching and identification of grey literature, in order to find papers that would be relevant to answering the above aim

2. To critically appraise and extract relevant data from all papers selected for inclusion in the review
3. To review and synthesise the evidence available, taking into account the quality of the data, to come to a conclusion about whether the proportion of young adults who consume alcohol has declined in recent years

Preliminary Literature Review

A preliminary review of the literature appears to suggest that the proportion of young adults who consume alcohol has decreased over time in many high-income countries. A UK-based study, for example, found the proportion of 18–24-year-old "non-drinkers" increased from 15% to 24% in 2005 to 2015. Similar trends have been seen in Italian, Swedish and Norwegian surveys although there is significant heterogeneity between countries with regards to the proportion of the young adult population who consume alcohol and the scale of the decline in recent years. Consumption by sex also varies. The Swedish survey found a greater increase in abstention and greater overall abstention rate in men compared to women, with the Norwegian study finding the reverse. Overall, this variation between countries suggests cultural factors may play a role in drinking habits.

In addition, a series of surveys from New Zealand found that the proportion of past year drinkers fell between 1995 to 2004 in the 18-19, 20-24 and 20-29 age groups. The 20-24 age group had the highest proportion of past year drinkers amongst the whole survey population (ages 14-65); this fell from 94.6% in 1995 to 89.3% in 2004. Further data from this survey suggests this decrease in proportion drinking is part of a wider change in drinking habits. Frequency of drinking declined over time in all three age groups, but the number of drinks consumed per drinking session increased.

These wider changes to drinking habits have also been documented elsewhere. In contrast to the above studies, papers from Finland and the United States (US) found an increase in the proportion of young adult alcohol consumers. In the US, the proportion of 18–24-year-olds who had consumed alcohol in the past year increased from 70.8% to 78.0% between 2001-2 and 2012-13. Overall, however, those who did drink alcohol drank less frequently and drank less per session than previous cohorts. Further research from the US initially appears at odds with this, with binge drinking increasing in the 21–23-year-old age group for both men and women. One potential explanation is the differences in age groups between the studies, with a decline in binge drinking amongst 18–20-year-olds in the initial study offsetting the rise of binge drinking amongst 21-24-year-olds. This could indicate that legal age of alcohol consumption, which is 21 years in the US, may influence these changes to wider trends in alcohol consumption.

Proposed Research Strategy

This project will be a systematic review of available literature. As this review involves the use of prevalence data, the condition-context-population (CoCoPop) mnemonic will be used as suggested by the Joanna Briggs Institute (Table 1). The geographical regions chosen have been selected as previous systematic reviews have demonstrated a decline in adolescent drinking in these areas. A time range ensuring at least some data is included from after the millennium shift was included to ensure that more recent drinking trends are captured. There is no clear definition of "young adulthood" – the age range of 18-30 years as this has precedent in other literature and encompasses the ages identified in the preliminary literature review.

Element	Description	Key Concept for this Systematic Review
**Co**ndition	Variable of interest	Proportion of the population who consume alcohol

		(Search terms will also be used to search for the inverse – proportion of the population who do not consume alcohol)
**Co**ntext	Context or specific setting such as geographical and time parameters	*Geography:* High-income countries in Europe, North America and Australasia which have seen a decline in adolescent drinking<br>Europe: Belgium, Bulgaria, Croatia, Czech Republic, Estonia, Finland, France, Germany, Greece, Hungary, Iceland, Ireland, Italy, Lithuania, the Netherlands, Norway, Poland, Portugal, Russia, Sweden, Switzerland and the United Kingdom<br>North America: USA and Canada<br>Australasia: Australia and New Zealand<br><br>*Time:* inclusion of at least one survey measurement since the year 2000
**Pop**ulation	Appropriate population	Young adults (aged 18-30 years)

Table 1. Condition-Context-Population description of research question.

Search Strategy

Three databases will be searched: Ovid MEDLINE, Ovid PsycINFO and the Cumulative Index for Nursing and Allied Health (CINAHL). Searches will include key details such as alcohol use, age of participants (youth), temporal changes and the countries previously identified. The full planned search strategy is outline below (Table 2).

Additionally, the bibliography of all included studies will be examined to determine if they also meet the inclusion and exclusion criteria. Forward searches will be conducted using Google Scholar, with the top 20 citations for each included study being reviewed to determine if they are eligible for inclusion. To identify grey literature, Google and Google Scholar searches will be carried out using terms such as "[country] + trend + young adult + alcohol + survey". The first 20 results for each search will be reviewed to determine if they meet the inclusion and exclusion criteria. Ideally, multiple leading authors in the field would be

contacted and hand-searching of selected journals would be performed, however this is unlikely to be feasible within the time constraints of this dissertation submission.

Concept	Database Heading	Free Text
Young adult	Young adult	youth* or young person* or young people or college student* or university student* or young adult* or emerg* adult* or early adult*.ti,ab
Alcohol consumption	Exp drinking behaviour<br>Exp alcohol drinking<br>Alcoholism<br>Alcohol intoxication	alcohol*<br>alcoholic beverage*<br>heavy adj3 drink*<br>bing* adj3 (drink* or consum* or intoxicat*)<br>non?drink* or abstain*
Trends over time	Longitudinal study	Trend* or declin* or time?serie* or temporal change* or longitudinal* or cohort* or wave* or (repeat* adj2 survey*) or chang* over time or trajector*.ti,ab
Countries of interest	exp Canada/ or exp United States/ or Belgium/ or Estonia/ or Lithuania/ or Bulgaria/ or Croatia/ or Czech Republic/ or Hungary/ or Poland/ or exp Russia/ or France/ or exp Germany/ or exp Gibraltar/ or exp United Kingdom/ or Greece/ or Ireland/ or Netherlands/ or Portugal/ or exp Denmark/ or Finland/ or Iceland/ or exp Sweden/ or Switzerland/ or exp Australia/ or New Zealand/	Belgium or Bulgaria or Croatia or Czech Republic or Denmark or Estonia or Finland or France or Germany or Greece or Hungary or Iceland or Ireland or Italy or Lithuania or Netherlands or Norway or Poland or Portugal or Russia or Sweden or Switzerland or United Kingdom or UK or England or Scotland or Wales or Northern Ireland or US or USA or United States or Canada or Australia or New Zealand.ti,ab
Time frame	Limit search to 2000 to current	
NOT		Clinical* or *patient or *patients or patient* or disease* or pathol* or ward* or department* or mortal*.ti,ab<br>Pre?natal or birth* or new?born*.ti,ab<br>Cancer*.ti<br>Human immunodeficiency virus or HIV*.ti,ab<br>Mental health* or mental illness* or psych* or suicid*.ti

	Qualitative interviews<br>Interview as a topic	qualit* interview*.kw<br>latent adj2 analysis.ti,ab

Table 2. Planned search strategy. Terms within the same concept will be combined with "OR" and different concepts will be combined with "AND".

Data Extraction

Firstly, once all initial searches are complete, references will be exported into reference management software and duplicates discarded. Titles and abstracts will be then screened by one reviewer to ensure that are relevant to the research question and they meet the inclusion and exclusion criteria shown below (Table 3). A minimum of a two-year time span has been chosen to ensure trend over time can be measured. "English language" has been chosen as a criterion due to the time constraints of this review and lack of funding; this will be acknowledged as a limitation.

Ideally, two reviewers would screen all abstracts independently, to ensure inclusion criteria are consistently applied and to minimise the risk of randomly missing a paper. The constraints of this dissertation, however, means that this approach is not possible. Where information provided in the title and/or abstract is insufficient, the full text of the article will be downloaded. The precision of the search strategy will be assessed by dividing the number of included studies by the total number of potential articles identified.

Inclusion Criteria	Exclusion Criteria
• Age range: 18-30<br>• Shows at least two time points explicitly, one of which must be 2000 or later<br>• At least 2-year time span	• Special populations (e.g. homeless)

• English language	

Table 3. Inclusion and exclusion criteria for papers.

Data will be quality assessed and extracted into a data extraction form, which will first be piloted on several studies. The following data will be extracted for each article:

- Author(s) and year of publication
- Country or countries included
- Number of participants
- Age range of participants
- Time period for data collection
- Data on primary outcome: measure used to determine 'current alcohol drinker'; proportion of young adult population who drink for each study time period given; general description of trend over time
- Data on any relevant secondary outcomes – for example, measure and associated proportion of binge drinkers
- Information on funding sources and any conflicts of interest

Where data is missing, this will be commented upon. Depending upon time constraints of this dissertation, a single attempt may be made to reach authors for clarification. Once data has been extracted, it will be compiled into three tables: a list of all included studies, a summarised data extraction table of all studies and a critical appraisal review table.

Outcomes

The primary outcome is to determine if there has been a change in the proportion of the young adult population who consume alcohol over time, in selected high-income countries which have seen a decline in adolescent drinking.

This outcome has been chosen as a reflection of "overall" drinking habits, as a simple division into 'drinkers' and 'non-drinkers'. It is acknowledged as a limitation that some research will choose to measure alcohol consumption in other ways (such as per-capita consumption) and thus will not be included within this review. Data will be collected on relevant secondary measures, such as the proportion of the population who binge drink. If sufficient numbers of studies focusing on these outcomes are identified, they will be presented as a subset analysis.

Risk of Bias

All studies selected for inclusion will be assessed for risk of bias using the Joanna Briggs Institute critical appraisal checklist for studies reporting prevalence data. It is unlikely that any studies will be excluded on the basis of poor performance on this appraisal, but the results will be used to inform the data synthesis. A critical appraisal performance table will be included in the final dissertation output.

Data Synthesis

The expected output of this review is a narrative synthesis, given that data are likely to be heterogenous with regard to age range and time period. The narrative synthesis will be supported with a table, likely a simplified version of the data extraction table such as Table 4. At present, no specific subgroup analyses are planned. Results will be displayed by

geographical region. Should a suitable subset of studies be identified (for example, based on age range), then quantitative analysis may be performed.

Author	Country	Age Range	Time Range of Study	Overall Trend
				↗ (increase over time)<br>↘ (decrease over time)<br>↔ (no change over time)

Table 4. Example table to display findings for the primary outcome of the dissertation.

Project Plan

Potential Issues

There are no ethical issues anticipated with this project. Following the advised risk assessment, this project has been deemed as low risk. A completed risk assessment form is attached.

There may be some issues about practical access to studies. Any studies which are not immediately available as full-text via the University of Sheffield will be requested via the School of Health and Related Research Library's inter-library request service. Additionally, this is the author's first time conducting a systematic review and consequently there may be times when guidance is required. Consequently, the author is undertaking the HAR6501 Systematic Reviews and Critical Appraisal Techniques module to gain more knowledge about the subject. Additionally, they will seek guidance from their supervisor when required and, for appropriate queries, the University of Sheffield Library Help service.

Proposed Time Frame

Action	Anticipated Date For Completion	Expected Time Frame	2021								
			Jan	Feb	Mar	Apr	May	Jun	Jul	Aug	Sept
Supervisor meetings	Ongoing	Length of dissertation project									
1. Initial scoping searches	17th Feb 2021	2 weeks									
2. Define research question and develop an appropriate search strategy	3rd March 2021	2 weeks									
3. Write and submit research proposal	17th March 2021	2 weeks									
4. Present to Sheffield Alcohol Research Group for support and critique of search strategy	7th April 2021	1 day									
5. Draft introduction chapter	31st May 2021	2 weeks									
6. Conduct database searches	23rd Jun 2021	2 days									
7. Select studies for inclusion in research	2nd July 2021	1 week									
8. Conduct quality assessment of studies	9th July 2021	1 week									
9. Data extraction	23rd July 2021	1 week									
10. Data synthesis	6th August 2021	2 weeks									
11. Draft methodology, results and discussion/conclusion chapters	16th August 2021	4 weeks									
12. Send full draft submission for review	18th August 2021	1 week									
13. Final edits/view	7th September 2021	2 weeks									
14. Submit dissertation	8th September	1 day									

Table 5. Gantt Chart showing actions and proposed time scale for completing the research project.[12, 14]

List of Changes from Initial Protocol

Change	Comment
Narrowing of focus to English-speaking countries	I discussed this with my supervisor after initial scoping searches identified a high proportion of material from grey literature sources. A joint decision was made to narrow the focus to English-speaking countries as my lack of language skills and the time constraints of this review meant that I would not be able to include grey literature sources from non-English-speaking countries.
Clarification of age categories (addition of 16-34 age range)	I identified that some sources of data reported age ranges as, for example, 16-24 and 25-34. A tight age range of 18-30 would not allow include for either set of data, therefore a decision was made to expand the range to 16-34 years but to ensure that at least some participants were in the "core" 18-30 age range.
Division of general population into 18-25, 18-25 and 16-24 age ranges	Separated into three separate groups to allow for a more coherent description of the results.
Increase in the number of citations searched per record	Citation searches were increased from 20 to 50 to try and ensure a broader scope of grey literature coverage.
List of grey literature sources developed	Initially, my plan was to conduct a Google search of "[country] + young adult + alcohol + survey" as part of grey literature searching, and use the first 20 (increased to 50, as above) results as potential sources. Instead, this search was additionally refined to create a list of potential grey literature sources. This list was then was reviewed by an expert in the field to create a final list of grey literature searches.
Slight alterations to search strategy	Following discussion with a Health Sciences librarian and members of the Sheffield Alcohol Research Group (SARG), several recommendations were made to amend the search terms used to broaden the scope as needed. This generally included removal of some of the "NOT" terms and advice around how to best to search for country-level data. Additionally, non-English speaking countries were removed from the search for reasons discussed above.
Change from 2-year to 3-year time span and all time points from 2000 onwards	It was difficult to determine the length of time required for a change in prevalence to be considered a trend. This initial protocol considered a 2-year change to be acceptable. Discussion with members of SARG, however, suggested an increase to 3 years may be more appropriate and the decision was taken to change this. Similarly, discussion with team members meant a move to ensuring all data points were after the decline in adolescent drinking was a more appropriate choice.
Greater clarification of population eligibility criterion	After discussion with my supervisor, I decided the population eligibility criterion was slightly vague. This was clarified further in the review eligibility criteria table, with reasoning for population choices discussed in the text.

Addition of survey type and source data to eligibility criteria	The initial inclusion and exclusion criteria did not specify the study type or source data which would be considered eligible. This was rectified for the review article. Data from longitudinal cross-sectional surveys was required to assess trends over time. Limitations were placed on type of source data to exclude less relevant sources such as systematic reviews.
Removal of secondary outcomes from data collection	Initially, I planned to collect data on relevant secondary outcomes such as the proportion of binge drinkers. Given the time restraints of this student assignment, I made the decision to focus solely on the primary outcome instead.

Supplementary Material 2 – Search Strategies

Ovid MEDLINE(R) and Epub Ahead of Print, In-Process, In-Data-Review & Other Non-Indexed Citations and Daily <1946 to April 21, 2021>

Line	Search String	Results
1	Young Adult/	909673
2	(youth* or young person* or young people or college student* or university student* or young adult* or emerg* adult* or early adult*).ti,ab.	239420
3	1 or 2	1089486
4	exp drinking behavior/ or exp alcohol drinking/	77757
5	Alcoholism/	76113
6	Alcoholic Intoxication/	12604
7	(alcohol* or alcoholic beverage* or non?drink* or abstain*).mp.	443395
8	(bing* adj3 (drink* or consum* or intoxicat*)).mp.	6884
9	((heavy or moderate or light) adj3 drink*).mp.	11511
10	4 or 5 or 6 or 7 or 8 or 9	451546
11	Longitudinal Studies/	144033
12	(Trend* or time?serie* or temporal change* or longitudinal* or wave* or (repeat* adj3 (survey* or stud*)) or chang* over time or trajector*).ti,ab.	1176588
13	11 or 12	1225946
14	3 and 10 and 13	7161
15	exp Canada/ or exp United States/ or exp United Kingdom/ or Ireland/ or exp Australia/ or New Zealand/	2064254
16	(Ireland or United Kingdom or UK or Great Britain or England or Scotland or Wales or Northern Ireland or US or USA or United States or Canada or Australia or New Zealand).ti,ab,ia	1259484
17	15 or 16	2829212
18	14 and 17	2972
19	Qualitative Research/	61496
20	Interviews as Topic/	64816
21	(qualit* adj2 interview*).kw.	764
22	(latent adj2 analys*).ti,ab.	7789
23	19 or 20 or 21 or 22	120269
24	(Clinical* or patient* or inpatient* or outpatient* or pathol* or ward* or department*).ti,ab.	9325370
25	(Pre?natal or birth* or new?born*).ti.	190169
26	(Cancer or human immunodeficiency virus or HIV*).ti.	1269443
27	24 or 25 or 26	10052642
28	18 not (23 or 27)	2192
29	limit 28 to yr="2000 -Current"	2027

APA PsycInfo <1987 to April Week 2 2021>

Line	Search String	Results
1	Young Adult/	3756
2	(youth* or young person* or young people or college student* or university student* or young adult* or emerg* adult* or early adult*).ti,ab.	241947
3	1 or 2	242180
4	exp drinking behavior/ or exp alcohol drinking/	77757
5	Alcoholism/	22655
6	Alcoholic Intoxication/	2262
7	(alcohol* or alcoholic beverage* or non?drink* or abstain*).mp.	130551
8	(bing* adj3 (drink* or consum* or intoxicat*)).mp.	5669
9	((heavy or moderate or light) adj3 drink*).mp.	7650
10	4 or 5 or 6 or 7 or 8 or 9	133629
11	Longitudinal Studies/	12501
12	(Trend* or time?serie* or temporal change* or longitudinal* or wave* or (repeat* adj3 (survey* or stud*)) or chang* over time or trajector*).ti,ab.	262866
13	11 or 12	268649
14	3 and 10 and 13	3849
15	(Ireland or United Kingdom or UK or Great Britain or England or Scotland or Wales or Northern Ireland or US or USA or United States or Canada or Australia or New Zealand).lo.	901637
16	(Ireland or United Kingdom or UK or Great Britain or England or Scotland or Wales or Northern Ireland or US or USA or United States or Canada or Australia or New Zealand).ti,ab,ia	392733
17	15 or 16	1067675
18	14 and 17	2482
19	Qualitative Methods/	9473
20	Interviews/	8799
21	(qualit* adj2 interview*).ab.	12400
22	(latent adj2 analys*).ti,ab.	7783
23	19 or 20 or 21 or 22	37191
24	(Clinical* or patient* or inpatient* or outpatient* or pathol* or ward* or department*).ti,ab.	1005137
25	(Pre?natal or birth* or new?born*).ti.	18409
26	(Cancer or human immunodeficiency virus or HIV*).ti.	70774
27	24 or 25 or 26	1056669
28	18 not (23 or 27)	2110
29	limit 28 to yr="2000 -Current"	2013

CINAHL

Line	Search String	Results
S1	MH "Young adult"	259398
S2	TI (youth* or young person* or young people or college student* or university student* or young adult* or emerg* adult* or early adult*)	67602
S3	AB (youth* or young person* or young people or college student* or university student* or young adult* or emerg* adult* or early adult*)	117348
S4	S1 or S2 or S3	373629
S5	(MH "Drinking behavior+") or (MH "Alcohol drinking+")	33647
S6	MH "Alcoholism"	16342
S7	MH "Alcoholic intoxication"	3327
S8	(alcohol* or alcoholic beverage* or ((heavy or light or moderate) N3 drink*) or (bing* N3 (drink* or consum* or intoxicat*)) or (risk* N3 (drink* or consum*)) or non?drink* or abstain*)	115487
S9	S5 or S6 or S7 or S9	116335
S10	S4 and S9	20423
S11	MH "Prospective studies"	464567
S12	TI (trend* or time?serie* or temporal change* or longitudinal* or wave* or (repeat* N3 (survey* or stud*)) or chang* over time or trajector*)	76950
S13	AB (trend* or time?serie* or temporal change* or longitudinal* or wave* or (repeat* N3 (survey* or stud*)) or chang* over time or trajector*)	235390
S14	S11 or S12 or S13	661522
S15	S10 and S14	5305
S16	(MH "Canada+") or (MH "United States+") or (MH "United Kingdom+") or (MH "Ireland") or (MH "Australia+") or (MH "New Zealand")	1287186
S17	TI ((Ireland or United Kingdom or UK or Great Britain or England or Scotland or Wales or Northern Ireland or US or USA or United States or Canada or Australia or New Zealand)) or AB ((Ireland or United Kingdom or UK or Great Britain or England or Scotland or Wales or Northern Ireland or US or USA or United States or Canada or Australia or New Zealand)) or AF ((Ireland or United Kingdom or UK or Great Britain or England or Scotland or Wales or Northern Ireland or US or USA or United States or Canada or Australia or New Zealand))	1931186
S18	S16 or S17	2708302
S19	S15 and S18	3683
S20	(MH "Qualitative studies") or (MH "Interviews")	236507
S21	AB qualit* N2 interview* OR TI latent N2 analys* or AB latent N2* analys*	17545
S22	S20 or S21	244913
S23	TI ((clinical* or *patient or *patients or patient* or pathol* or ward* or department*)) or AB ((clinical* or *patient or *patients or patient* or pathol* or ward* or department*))	2337763

S24	TI (pre?natal or birth* or new?born*)	35844
S25	TI (cancer or human immunodeficiency virus or HIV*)	357171
S26	S23 or S24 or S25	2551484
S27	S19 not (S22 or S26)	2532
S28	S19 not (S22 or S26) – limiters published date 20000101-20211231	2472

Supplementary Material 3 – Grey Literature Sources

Country	Type of Source	Source
Europe		
UK	Charity	Alcohol Change UK https://alcoholchange.org.uk/research-hub/headline-reports
UK	Charity	Balance North East http://www.balancenortheast.co.uk/
UK	Charity	Institute for Alcohol Studies https://www.ias.org.uk/
UK (Scotland)	Charity	Alcohol Focus Scotland https://www.alcohol-focus-scotland.org.uk/resources/
UK (Scotland)	Charity	Scottish Health Action for Alcohol Problems https://www.shaap.org.uk/our-work/alcohol-young-people.html
UK (Northern Ireland)	Research Centre (Govt Website)	Public Health Agency https://www.publichealth.hscni.net/publications?keys=alcohol
UK (Great Britain)	Survey	ONS – Opinion and Lifestyle Survey https://www.ons.gov.uk/peoplepopulationandcommunity/healthandsocialcare/drugusealcoholandsmoking/datasets/adultdrinkinghabits
UK (England)	Survey	Health Survey for England http://healthsurvey.hscic.gov.uk/data-visualisation/data-visualisation/explore-the-trends/alcohol.aspx
UK (Northern Ireland)	Survey	Adult Drinking Patterns Survey https://www.health-ni.gov.uk/articles/adult-drinking-patterns-survey
UK (Northern Ireland)	Survey	Continuous Household Survey https://www.nisra.gov.uk/statistics/find-your-survey/continuous-household-survey
UK (Scotland)	Survey	Scottish Health Survey https://www.gov.scot/publications/scottish-health-survey-2019-volume-1-main-report/
UK (Wales)	Survey	National Survey for Wales https://gov.wales/national-survey-wales
UK (Wales)	Survey	Higher Education Alcohol and Drug Survey https://criminology.research.southwales.ac.uk/substance-use-research-group/research-projects/
Ireland	Charity	Alcohol Action Ireland https://alcoholireland.ie/policy/

Ireland	Research Centre (Govt Website)	Health Research Board https://www.drugsandalcohol.ie/key-irish-data/
Ireland	Govt Advisory Board	National Advisory Committee on Drugs and Alcohol https://www.nacda.ie/
Ireland	Survey	Drug Prevalence Survey https://www.nacda.ie/index.php/publications.html
North America		
US	Research Centre	Alcohol Research Group https://arg.org/research/overview/
US	Research Centre	National Institute on Alcohol Abuse and Alcoholism https://www.niaaa.nih.gov/research
US	Research Centre	National Institute on Drug Abuse https://www.drugabuse.gov/research
US	Survey	Behavioural Risk Factor Surveillance System https://www.cdc.gov/brfss/data_documentation/index.htm
US	Survey	Monitoring the Future http://www.monitoringthefuture.org/
US	Survey	National Alcohol Survey https://arg.org/center/national-alcohol-surveys/
US	Survey	National Epidemiological Survey on Alcohol and Related Conditions (NESARC) https://www.niaaa.nih.gov/research/nesarc-iii
US	Survey	National Health Interview Survey https://www.cdc.gov/nchs/nhis/index.htm
US	Survey	National Health and Nutrition Examination Survey (NHANES) https://www.cdc.gov/nchs/nhanes/nhanes_products.htm
US	Survey	National Longitudinal Survey of Youth https://www.nlsinfo.org/
US	Survey	National Survey on Drug Use and Health https://nsduhweb.rti.org/respweb/homepage.cfm
US	Survey	Youth Risk Behaviour Surveillance System (12th grade) https://www.cdc.gov/healthyyouth/data/yrbs/overview.htm
Canada	Charity (overseen by Health Canada)	Canadian Centre on Substance Use and Addiction https://www.ccsa.ca/publications

Canada	Research Centre	Canadian Institute for Substance Use Research https://www.uvic.ca/research/centres/cisur/publications/index.php
Canada	Survey	Canadian Alcohol and Drug Use Monitoring Survey https://www.canada.ca/en/health-canada/services/health-concerns/drug-prevention-treatment/canadian-alcohol-drug-use-monitoring-survey.html
Canada	Survey	Canadian Community Health Survey https://www.canada.ca/en/health-canada/services/food-nutrition/food-nutrition-surveillance/health-nutrition-surveys/canadian-community-health-survey-cchs.html
Canada	Survey	Canadian Student Tobacco, Alcohol and Drugs Survey https://uwaterloo.ca/canadian-student-tobacco-alcohol-drugs-survey/
Canada	Survey	Canada Tobacco, Alcohol and Drugs Survey https://www.canada.ca/en/health-canada/services/canadian-tobacco-alcohol-drugs-survey/2017-summary.html
Australasia		
Australia	Charity	Foundation for Alcohol Research and Education https://fare.org.au/research/
Australia	Research Centre	National Centre for Education and Training on Addiction https://nceta.flinders.edu.au/
Australia	Research Centre	National Drug and Alcohol Research Centre https://ndarc.med.unsw.edu.au/our-projects
Australia	Research Centre	National Drug and Alcohol Research Institute https://ndri.curtin.edu.au/publications-resources
Australia	Survey	Australian Temperament Project https://www.melbournechildrens.com/atp/
Australia	Survey	Australian Longitudinal Study on Women's Health https://alswh.org.au/
Australia	Survey	HILDA (Household, Income and Labour Dynamics in Australia) Survey https://melbourneinstitute.unimelb.edu.au/hilda/publications
Australia	Survey	National Drug Strategy Alcohol Survey https://www.aihw.gov.au/about-our-data/our-data-collections/national-drug-strategy-household-survey
Australia	Survey	Ten to Men: Australian Longitudinal Study on Male Health https://tentomen.org.au/

New Zealand	Charity	Alcohol.org.nz<br>https://www.alcohol.org.nz/resources-research/alcohol-research/research-and-information-publications
New Zealand	Research Centre	Tea Hiringa Haurora (Health Protection Agency)<br>https://www.hpa.org.nz/our-work/research/publications
New Zealand	Research Centre	Massey University, SHORE and Whāriki Research Centre<br>https://shoreandwhariki.ac.nz/alcohol-1
New Zealand	Survey	New Zealand Health Survey<br>https://www.health.govt.nz/nz-health-statistics/health-statistics-and-data-sets/alcohol-use-data-and-stats
New Zealand	Survey	Youth 2000 Series (age group 17+)<br>https://www.youth19.ac.nz/publications

Supplementary Material 4 – Survey Results with Time Spans under 10 Years

Country	Survey	Age Range (Years)	Years	Measure	Prevalence (%)	% Change	Trend
**18-25**							
Canada	Canadian Alcohol and Drug Use Monitoring Survey (CADUMS) (Government of Canada, 2020)	20-24	2008-2012	Past month	T: 72.3 to 66.1	-6.2	↘
				Past year	T: 84.5 to 83.7	-0.8	↔
	Canadian Tobacco, Alcohol and Drugs Survey (Government of Canada, 2016, 2021)	20-24	2013-2017	Past month	T: 73.9 to 71.3	-2.7	↔
				Past year	T: 83.2 to 83.5	+0.3	↔
				Lifetime	T: 90.4 to 88.3	-2.1	↔
UK (Northern Ireland)	Continuous Household Survey (NISRA, 2017; Smith & Foxcroft, 2009)	18-24	2000/1-2008/9	"Nowadays"	T: 82 to 87<br>M: 88 to 87<br>F: 79 to 87	+5<br>-1<br>+8	T: ↔<br>M: ↔<br>F: ↗
UK (Wales)	National Survey for Wales (Murphy, 2021)	18-24	2016/17-2019/20	Past year	T: 87 to 85	-2	↔
New Zealand	New Zealand Health Survey	18-24	2011/12-2018/19 (Ministry of Health, n.d.b)	Past year	M: 88.5 to 87.7<br>F: 81.0 to 83.5	-0.8<br>+2.5	↔
	New Zealand National Alcohol Survey (Huckle et al., 2011)	18-19	2000-2004	Past year	M: 91.7 to 89.3<br>F: 89.1 to 87.5	-2.4<br>-1.6	↔
		20-24			M: 94.4 to 89.3<br>F: 87.0 to 89.4	-5.1<br>+2.4	M: ↘<br>F: ↔
**18-35**							
UK (England)	Health Survey for England (NHS Digital, 2020)	25-34	2011-2019	Past year[b]	T: 84 to 80<br>M: 87 to 85<br>F: 81 to 76	-4<br>-2<br>-5	↔

UK (Northern Ireland)	Adult Drinking Patterns in Northern Ireland	18-29	2008-2013 (Central Survey Unit, 2008; Information Analysis Directorate, 2014)	Past week	M: 73 to 62<br>F: 57 to 40	-11<br>-17	↘
			2005-2013 (Central Survey Unit, 2006; Information Analysis Directorate, 2014)	Past year	T: 86 to 82<br>M: 86 to 86<br>F: 86 to 79	-4<br>0<br>-7	T: ↔<br>M: ↔<br>F: ↘
UK (Wales)	National Survey for Wales (Murphy, 2021)	18-30	2016/17-2019/20	Past year	T: 87 to 83<br>M: 87 to 84<br>F: 86 to 82	-4<br>-3<br>-4	↔
		25-30	2016/17-2019/20	Past year	T: 86 to 81	-5	↔
Ireland	Irish Health Survey (Central Statistics Office, 2016, 2020)	25-34	2015-2019	Past year	T: 89 to 84	-5	↔
New Zealand	New Zealand Health Survey	25-34	2011-12-2018/19 (Ministry of Health, n.d.b)	Past year	M: 87.8 to 87.6<br>F: 77.7 to 76.9	-0.2<br>-0.8	↔
	New Zealand National Alcohol Survey	25-29	2000-2004 (Huckle et al., 2011)	Past year	M: 89.5 to 87.8<br>F: 82.1 to 84.9	-1.7<br>+2.8	↔
**16-24**							
UK (England)	Health Survey for England	16-24	2011-2019 (NHS Digital, 2020)	Past year[b]	T: 81 to 72<br>M: 82 to 74<br>F: 79 to 69	-9<br>-8<br>-10	↘

UK (Northern Ireland)	General Household Survey (Smith & Foxcroft, 2009)	16-24	2000-2006	Past week	M: 70 to 60<br>F: 64 to 53	-10<br>-11	↘

Key:
- ↗ Increase of >5.0% between the start and end point provided
- ↘ Decrease of >5.0% between the start and end point provided
- ↔ Stable – any value with an increase or decrease of ≤5% between the start and end point provided

Abbreviations used: UK = United Kingdom, T = total, M = male, F = female
[a] Author-calculated value based on data available in the original record.
[b]Reported in record as abstention data.

References for Supplementary Material 4:

Government of Canada. (2016). *Canadian Tobacco, Alcohol and Drugs Survey (CTADS) 2013: supplementary tables*. https://www.canada.ca/en/health-canada/services/canadian-alcohol-drugs-survey/2013-supplementary-tables.html

Government of Canada. (2020). Alcohol use among Canadians. https://health-infobase.canada.ca/alcohol/ctads/

Government of Canada. (2021). *Canadian Tobacco, Alcohol and Drugs (CTADS) Survey: 2017 detailed tables.* https://www.canada.ca/en/health-canada/services/canadian-alcohol-drugs-survey/2017-summary/2017-detailed-tables.html.

North Ireland Statistics and Research Agency (NISRA). (n.d.). *Continuous Household Survey.* https://www.nisra.gov.uk/statistics/find-your-survey/continuous-household-survey.

Smith, L., & Foxcroft, D. (2009). *Drinking in the UK: An exploration of trends.* Joseph Rowntree Foundation. https://www.jrf.org.uk/report/drinking-uk-exploration-trends

Murphy, R. (Knowledge and Analytical Services, Welsh Government). (2021). Email file entitled "NSW – Non Drinker breakdowns.xlsx" received by JD on 9 Jul 2021. Initial contact to surveys@gov.wales made by JD on 1 Jul 2021.

Ministry of Health. (n.d.b) *Indicator: Past-year drinkers (had alcoholic drink in the past 12 months).* https://minhealthnz.shinyapps.io/nz-health-survey-2020-21-annual-data-explorer/_w_8811e512/#!/explore-indicators

Huckle, T., You, R. Q., & Casswell, S. (2011). Increases in quantities consumed in drinking occasions in New Zealand 1995-2004. Drug and Alcohol Review, 30, 366-371. https://doi.org/10.1111/j.1465-3362.2010.00220.x

NHS Digital. (2020). *Health Survey for England, 2019: data tables.* https://digital.nhs.uk/data-and-information/publications/statistical/health-survey-for-england/2019/health-survey-for-england-2019-data-tables

Central Survey Unit. (2008). *Adult Drinking Patterns in Northern Ireland 2008.* Department of Health, Social Services and Public Safety. https://www.health-ni.gov.uk/sites/default/files/publications/dhssps/adps-2008.pdf

Information Analysis Directorate. (2014). *Adult Drinking Patterns in Northern Ireland 2013*. Department of Health, Social Services and Public Safety. https://www.health-ni.gov.uk/sites/default/files/publications/dhssps/adps-2013.pdf

Central Survey Unit. (2006). *Adult Drinking Patterns in Northern Ireland 2005*. Department of Health, Social Services and Public Safety. https://www.health-ni.gov.uk/sites/default/files/publications/dhssps/adps-2005.pdf

Central Statistics Office. (2016). *Irish Health Survey 2015*. Cork, Central Statistics Office; https://www.cso.ie/en/releasesandpublications/ep/p-ihs/irishhealthsurvey2015/ct/

Central Statistics Office. (2020). *Irish Health Survey 2019 – main results*. Cork, Central Statistics Office. https://www.cso.ie/en/releasesandpublications/ep/p-ihsmr/irishhealthsurvey2019-mainresults/

Supplementary Material 5 – Record-Level Data

Record	Country	Population	Age Group[a]	Survey	Years	Measure	Start Year (%, with 95% CI or SE)	End Year (% with 95% CI or SE)	Statistically Significant?
Adult Drinking Patterns in Northern Ireland (2005 report, Central Survey Unit, 2006; 2008 report, Central Survey Unit, 2008; 2013 report, Information Analysis Directorate, 2014)	UK (NI)	GP	18-29	ADPNI	2005-2013	Consumption, past year	T: 86<br>M:86<br>F:86	T: 82<br>M: 86<br>F: 79	NR
					2008-2013	Consumption, past week	M: 73<br>F: 57	M: 62<br>F: 40	NR
Australian National Health Survey (2002 report, ABS, 2002; 2017-18 data sheet, ABS, 2017-18)	Australia	GP	18-24	ANHS	2001-2017/8	Consumption, past week	T: 63.3[b]<br>M:68.3[b]<br>F:58.1[b]	T: 48.4 [b]<br>M: 52.6 [b]<br>F: 44.0 [b]	NR
						Consumption, past year	T: 90.7[b]<br>M: 91.6[b]<br>F: 90.0[b]	T: 83.8[b]<br>M:84.4[b]<br>F: 83.3[b]	
						Abstention, lifetime	T: 6.7[b]<br>M: 6.5[b]<br>F: 7.0[b]	T: 11.4<br>M: 10.6<br>F:12.0	
			25-34			Consumption, past week	T: 63.2[b]<br>M:73.0[b]<br>F:53.7[b]	T: 52.1[b]<br>M: 61.3[b]<br>F: 43.5[b]	

						Consumption, past year	T: 87.3[b] M:90.0[b] F:84.7[b]	T: 80.4[b] M: 88.3[b] F: 73.3[b]	
						Abstention, lifetime	T: 7.5[b] M:6.1[b] F:8.8 [b]	T: 11.6 M: 7.4 F: 16.2	
Callinan et al., 2017	Australia	GP	18-29	NDSHS	2001-2013	Abstention, lifetime	T: 13 (11.9-14.2)	T: 21 (19.4-22.7)	p <0.001
Canadian Alcohol and Drug Use Monitoring Survey (CADUMS)/ Canadian Tobacco, Alcohol and Drugs Survey [data sheet] (Government of Canada, 2020)	Canada	GP	20-24	CADUMS	2008-2012	Consumption, past month	T: 72.7	T: 66.1	NR
						Consumption, past year	T: 84.5	T: 83.7	
				CTADS	2013-2017	Consumption, past month	T: 73.9	T: 73.1	
						Consumption, past year	T: 83.2	T: 38.5	
Canadian Tobacco, Alcohol and Drugs Survey (2013 data sheet, Government of Canada, 2016; 2017 data sheet, Government of Canada, 2021)	Canada	GP	20-24	CTADS	2013-2017	Consumption, past year	T: 83.2 (80.4-85.9)	T: 83.5 (80.1-86.8)	NS (CI)^
						Consumption. lifetime	T: 90.4 (88.2-92.5)	T: 88.3 (84.9-91.6)	
Chen & Yoon, 2021 (report)	US	GP	18-20	NSDUH	2000-2019	Consumption, past month	T: 49.0 (SE 0.9)	T: 35.9 (SE 0.9)	NR

							M: 52.4 (SE 1.2)<br>F: 45.4 (SE 1.2)	M: 33.8 (SE 1.2)<br>F: 38.2 (SE 1.4)	
Continuous Household Survey [data sheet] (NISRA, 2017)	UK (NI)	GP	18-24	CHS	2000/1-2008/9	Consumption, "nowadays"	T: 82<br>M: 88<br>F: 79	T: 87<br>M: 87<br>F: 87	NR
Dawson et al., 2015	US	GP	18-24	NESARC	2001/2-2012/3	Consumption, past year	T: 70.8 (SE 1)	T: 78.0 (SE 0.8)	p <0.001
Grant et al., 2017	US	GP	18-29	NESARC	2001/2-2012/13	Consumption, past year	T: 73.1 (71.5-74.7)	T: 80.1 (78.8-81.3)	p<0.05
Grucza et al., 2018	US	GP	18-29	BRFSS	2002-2013	Consumption, past month[c]	T: 60.5 (SE 0.2)	T: 56.1 (SE 0.2)	NR
				NAS	2012-2013[d]	Consumption, past year[c]	T: 69.3 (SE 1.3)	T: 75.3 (SE 1.9)	
				NESARC	2001/2-2012/3	Consumption, past year[c]	T: 73.2 (SE 0.4)	T: 80.1 (SE 0.3)	
				NHANES	2001/2-2013/4	Consumption, past year[c]	T: 75.7 (SE 2.78)	T: 80.1 (SE 2.1)	
				NHIS	2002-2013	Consumption, past year[c]	T: 65.1 (SE 0.4)	T: 68.3 (SE 0.4)	
				NSDUH	2002-2013	Consumption, past year[c]	T: 78.2 (SE 0.2)	T: 78.7 (SE 0.3)	
Hasin et al., 2019	US	GP	18-20	NSDUH	2002-2017	Consumption, past month	T: 54.8 (SE 0.46)<br>M: 59.1 (SE 0.61)	T: 40.0 (SE 0.50)<br>M: 40.8 (SE 0.56)	p <0.001<br><br>p <0.001

							F: 51.4 (SE 0.64)[e]	F: 40.3 (0.69)[e]	p <0.001
Health Survey for England [2019 data sheet] (NHS Digital, 2020)	UK (England)	GP	16-24	HSE	2000-2019	Abstention, past week	T: 37<br>M: 33<br>F: 41	T: 60<br>M: 57<br>F: 63	NR
					2011-2019	Abstention, past year	T: 19<br>M: 18<br>F: 21	T: 28<br>M: 26<br>F: 31	
			25-34		2000-2019	Abstention, past week	T: 28<br>M: 23<br>F: 33	T: 49<br>M: 43<br>F: 55	
					2011-2019	Abstention, past year	T: 16<br>M: 13<br>F: 19	T: 20<br>M: 15<br>F: 24	
Huckle et al., 2011	New Zealand	GP	18-19	NZNAS	2000-2004	Consumption, past year	M: 91.7<br>F: 89.1	M: 89.3<br>F: 87.5	NS<br>NS
			20-24				M: 94.4<br>F: 87.0	M: 89.3<br>F: 89.5	p=0.0273<br>NS
			25-29				M: 89.5<br>F: 82.1	M: 87.8<br>F: 84.9	NS<br>NS
Irish Health Survey (2015 report, Central Statistics Office, 2016; 2019 report, Central Statistics Office, 2020)	Ireland	GP	25-34	IHS	2015-2019	Consumption, past year	T: 89	T: 84	NR
Livingston, 2015 (report)	Australia	GP	18-24	NDSHS	2001-2013	Abstention, lifetime	T: 7.5 (6.2-8.8)	T: 13.7 (11.1-15.7)	p<0.05

			25-29				T: 4.1 (3.1-5.1)	T: 10.8 (8.9-12.6)	$p<0.05$
McCabe et al., 2021	US	College	18-22	NSDUH	2002-2018	Abstention, past year	T: 20.0	T: 28.0	NR
McKetta & Keyes, 2019	US	GP	18—29	NHIS	2006-2018	Abstention, past year	M: 27[f]<br>F: 39[f]	M: 28[f]<br>F: 30[f]	NR
Mongan et al., 2021 (report)	Ireland	GP	25-34	INDAS	2002/3-2019/20	Abstention, past year	T: 9.0	T: 18.7	NR
National Drug Strategy Household Survey (a) [data sheet] (AIHW, 2020)	Australia	GP	20-29	NDSHS	2001-2019	Abstention, past year	T: 8.9 (7.7-10.1)	T: 22.0 (19.8-25.0)	$p<0.05$
National Drug Strategy Household Survey (b) [data sheet] (AIHW, 2021)	Australia	GP	18-24	NDSHS	2001-2019	Abstention, past year	T: 9.7	T: 20.8	NR Significance not reported across whole time period
			25-29				T: 8.8	T: 23.6	
National Survey on Drug Use and Health (report) (SAMHSA, 2020)	US	GP	18-25	NSDUH	2002-2019	Consumption, past month	T: 60.5	T: 54.3	$p<0.05$
National Survey for Wales [data sheet] (Murphy, 2021)	UK (Wales)	GP	18-30	NSW	2016/17-2019/20	Consumption, past year	T: 87 (85-89)<br>M: 87 (84-91)<br>F: 86 (84-89)	T: 83 (80-86)<br>M: 84 (78-89)<br>F: 82 (78-87)	NR

			18-24			Consumption, past year	T: 87 (84-91)	T: 85 (79-90)	
			25-30			Consumption, past year	T: 86 (83-89)	T: 81 (77-86)	
New Zealand Health Survey (2019a data sheet, Ministry of Health, n.d.a; 2019b data sheet, Ministry of Health, n.d.b)	New Zealand	GP	18-24	NZHS	2006/7-2018/19	Consumption, past year	T: 89.1	T: 85.6 (82.5-88.4)	Significance not reported across whole time period
					2011/12-2018/19		M: 88.5 (83.6-92.4) F: 81.0 (77.4-84.4)	M: 87.7 (83.2-91.3) F: 83.5 (78.7-87.7)	NS (Ci)^
			25-34		2006/7-2018/19		T: 86.7	T: 82.3 (80.0-84.5)	Significance not reported across whole time period
					2011/12-2018/19		M: 87.8 (84.0-91.0) F: 77.7 (74.1-81.0)	M: 87.6 (84.4-90.4) F: 76.9 (73.6-80.0)	NS (Ci)^
Ng Fat et al., 2018	UK (England)	GP	16-24	HSE	2005-2015	Abstention, past week	T: 35 (31.8-39.1)	T: 50 (45.3-54.5)	p <0.001

						Abstention, “nowadays"	T: 18 (15.5-21.5)<br>M: 16 (12.7-21.0)<br>F: 20 (16.4-24.8)	T: 29 (24.9-33.1)<br>M: 25 (19.4-30.6)<br>F: 33 (28.2-38.7)	p <0.001<br>NS<br>p <0.001
						Abstention, lifetime	T:9 (7.1-11.5)	T: 17 (13.2-21.1)	p <0.001
			18-24			Abstention, “nowadays"	T: 15 (12.0-18.0)	T: 24 (20.0-28.5)	p <0.001
Opinion and Lifestyle Survey [data sheet] (Office for National Statistics, 2018)	UK (Great Britain)	GP	16-24	OLS	2005-2017	Consumption, past week	T: 60<br>M: 64<br>F: 56	T: 47.9 (43.5-52.4)<br>M: 47.5 (40.8-54.2)<br>F: 48.3 (42.3-54.4)	NR
						Abstention, “nowadays”	T: 19.0 (17.4-20.5)<br>M: 17.4 (15.2-19.7)	T: 22.8 (19.1-26.6)	NS (CI)^<br>NS (CI)^<br>NS (CI)^

							F: 20.4 (18.2-22.6)	M: 21.8 (16.3-27.3)<br>F: 24.0 (18.8-29.2)	
	UK (England)					Consumption, past week	T: 60<br>M:64<br>F: 56	T: 50.0 (45.5-55.0)<br>M:51.3 (43.9-58.3)<br>F: 48.8 (42.2-55.4)	NR
						Abstention, "nowadays"	T: 19<br>M: 17<br>F: 21	T: 22.6 (18.5-26.8)<br>M: 21.5 (15.4-27.6)<br>F: 23.8 (18.1-29.4)	NR
Schoenborn et al., 2004 (report)/ Schoenborn, et al., 2013 (report)	US	GP	18-24	NHIS	1999-2001 to 2008-2010	Consumption, past year	T: 61.8 (0.73)<br>M: 67.1 (0.93)	T: 62.4 (0.83)<br>M:65.9 (1.14)	NR

							F: 56.6 (0.92)	F: 58.9 (1.14)	
						Abstention, lifetime	T: 33.5 (0.70) M: 29.3 (0.91) F: 37.6 (0.88)	T: 33.4 (0.83) M: 30.5 (1.12) F: 36.2 (1.12)	
Schulenberg et al., 2020 (report)	US	College	18-22[g]	MtF	2000-2019	Consumption, past month	T: 67.4	T: 62.2	
						Consumption, past year	T: 83.2	T: 77.6	
						Consumption, lifetime	T: 86.6	T: 79.2	
		GP	19-20			Consumption, past month	T: 59.1	T: 45.6	
						Consumption, past year	T: 79.7	T: 64.3	
			21-22			Consumption, past month	T: 70.5	T: 68.4	
						Consumption, past year	T: 86.2	T: 82.0	
			19-22			Consumption, past month	M: 67.8 F: 62.2	M: 56.4 F: 58.2	
			23-24			Consumption, past month	T: 71.5	T: 73.8	
						Consumption, past year	T: 87.2	T: 86.7	

			25-26			Consumption, past month	T: 68.7	T: 75.0	
						Consumption, past year	T: 84.2	T: 87.8	
			23-26			Consumption, past month	M: 76.2<br>F: 65.8	M: 74.9<br>F: 74.1	
Scottish Health Survey (2003 data sheet, Bromley et al., 2008; 2008 data sheet, The Scottish Government, 2009; 2010 data sheet, Scottish Health Survey, 2020)	UK (Scotland)	GP	16-24	SHeS	2008-2019	Abstention, past year	T: 7 (4.7-10.1)	T: 17 (11.9-22.3)	P< 0.05 (CI)
					2003-2019		M: 11<br>F: 10	M: 17<br>F: 17	NR
			25-34		2008-2019		T: 10 (7.6-12.8)	T: 12 (9.0-15.3)	NS (CI)^
					2003-2019		M: 7<br>F: 10	M: 8<br>F: 16	NR
Slater et al., 2015 (report)	US	GP	18-20	NSDUH	2002-2013	Consumption, past month	F: 48.1 (SE 0.9)	F: 43.6 (SE1.3)	NR
			21-25				F: 60.7 (SE 0.9)	F: 65.2 (SE1.0)	
Smith & Foxcroft, 2009 (report)	UK (NI)	GP	18-24	CHS	2001/2-2006/7	Consumption, "nowadays"	M: 88<br>F: 79	M: 91<br>F: 86	NR
	UK (GB)		16-24	GHS	2000-2006	Consumption, past week	M: 70<br>F: 64	M: 60<br>F: 53	
Twenge & Park, 2019	US	College	18-22[g]	MTF	2000 to 2015	Consumption, lifetime	T: 87[f]	T: 79[f]	NR
		GP	19-30				T: 89.5[f]	T: 86[f]	
White et al., 2015	US	GP	18-20	NSDUH	2002-2012	Consumption, past month	M:54.6<br>F: 48.1	M: 46.7<br>F: 45.0	p≤0.01<br>NS at p<0.01

						Abstention, lifetime	M: 17.6<br>F: 19.0	M: 25.8<br>F: 25.4	p≤0.01<br>p≤0.01
			21-25			Consumption, past month	M: 72.6<br>F: 60.6	M: 72.8<br>F: 65.4	NS at p<0.01<br>p≤0.01
						Abstention, lifetime	M:8.4<br>F: 11.6	M: 9.1<br>F: 9.9	NS at p<0.01<br>NS at p<0.01
			18-25			Consumption, past month	M: 65.2<br>F: 55.7	M: 62.6<br>F: 57.9	NS at p<0.01<br>p≤0.01
						Abstention, lifetime	M:12.2<br>F: 14.5	M: 15.6<br>F: 15.6	p≤0.01<br>NS at p<0.01

Abbreviations used: 95% CI = 95% confidence interval, SE = standard error, SD = standard deviation, NR = not reported, NS = not significant, T = total, M = male, F = female, GP = general population, US = United States of America, UK = United Kingdom, GB = Great Britain, NI = Northern Ireland. The survey abbreviations are consistent with those shown in Supplementary Material 6.

[a]Note that where groups are provided for university studies, the ages may be reported as a mean or percentage value, with a comma separating the age in the start year and age in the end year.
[b]Author-calculated value based on data available in the original record.
[c]Estimates are based on the fitted trend line for each survey, using all years of available data. These results were used to calculate the predicted average prevalence for each survey and each subgroup for the years 2002 and 2013.
[d]Note that the National Alcohol Survey takes place approximately every 5 years (2000, 2005, 2009/10, etc) and so did not have surveys in 2002 and 2013. The data here is based on a fitted trend line using all years of available data.
[e]Non-pregnant females only.
[f]Figure obtained from a graph.
[g]Age taken as “within the first four years of finishing high school” and thus would generally include those aged 18-22.

Supplementary Material 6 – Summary of Surveys

Survey	Country	Age Group	Number of Records[a]	Years Covered	Reported Prevalence		Measure		Survey Mode	Response Rate
					Total	M/F	Cons	Abs		
NATIONAL CROSS-SECTIONAL										
Behavioral Risk Factor Surveillance System (BRFSS)	US	18-29	1	2002-2013	GP		1M		Telephone CATI	2002: median 44.5%[b]<br>2013: median 46.4%[b]
Monitoring the Future (MTF)[c]	US	18-30	2	2000-2019	C/GP	S/GP	LT, 1Y, 1M		In person SAPQ (12th grade)<br>Postal SAPQ (19-30)<br>From 2018: 50% postal, 50% CAWI	2000 (19-32): average 77%[b]<br>2019 (19-30): 37.8% postal, 46.8% web[b]
National Alcohol Survey (NAS)	US	18-29	1	2000-2015	GP		1Y		Telephone CATI	Unclear[d]
National Epidemiological Survey on Alcohol and Related Conditions (NESARC)	US	18-29	3	2001/2-2012/3	GP	GP	1Y		In person CAPI	2001/2: 81.0%[b]<br>2012/3: 60.1%[b]
National Health and Nutrition	US	18-29	1	2001/2-2013/14	GP		1Y		In person CAPI	2001/2: 84%[b]<br>2013/4: 71%[b]

Examination Survey (NHANES)										
National Health Interview Survey (NHIS)	US	18-29	3	1999-2001-2018	GP		1Y	LT, 1Y	In person CAPI	2000: 72.1%[b]<br>2018: 53.1%[b]
National Survey on Drug Use and Health (NSDUH)	US	18-34	7	2000-2019	C/GP	GP	1Y, 1M	LT, 1Y	1991-1998: SA PAPI<br>1999 onwards: ACASI	2000: 73.8%[b]<br>2019: 64.9%[b]
General Household Survey (GHS)	UK (GB)	16-24	1	2000-2006		GP	1W		In person CAPI or telephone CATI (2000 onwards)	2000: 67%[b]<br>2006: 76%[b]
Health Survey for England (HSE)	UK (England)	16-34	2	2000-2019	GP	GP		LT, 1Y, 1W, NWD	In person CAPI	2000: Unclear[d]<br>2019: 55%[b]
Opinion and Lifestyle Survey (OLS)	UK (GB and England)	16-24	1	2005-2017	GP	GP	1W	NWD	Telephone CATI or online CASI (2017 only)	Unclear[d]
Scottish Health Survey (SHeS)	UK (Scotland)	16-34	1	2003-2019	GP			1Y	In person CAPI plus SAPQ element	2003: 60%[b]<br>2019: 49%[b]
National Survey for Wales (NSW)	UK (Wales)	18-30	1	2016/17-2019/20	GP	GP	1Y		In person CAPI + CASI	2016/17: 48.4%[b]<br>2019/20: 49.8%[b]
Adult Drinking Patterns in Northern Ireland (ADPNI)	UK (NI)	18-29	1	2005-2013	GP	GP	1Y, 1W		In person CAPI	2005: 64[b]<br>2013: 65%[b]

Continuous Household Survey (CHS)	UK (NI)	18-24	1	2001/2-2006/7	GP	GP	NWD		In person CAPI	Unclear[d]
Australian National Health Survey (ANHS)	Australia	18-34	1	2001-2017/8	GP	GP	1Y, 1W	LT	F2F (NOS)	2001: Unclear[d]<br>2017: 76.1%[b]
National Drug Strategy Household Survey (NDSHS)	Australia	18-29	3	2001-2019	GP			LT, 1Y	F2F (NOS), telephone CATI or SAPQ ('drop and collect'), CAWI (2016 only)	2001:50%[b]<br>2019: 49%[b]
Canadian Alcohol and Drug Use Monitoring Survey (CADUMS)	Canada	20-24	1	2008-2012	GP		1Y, 1M		Telephone CATI	2008: Unclear[d]<br>2012: 39.5%
Canadian Tobacco, Drugs and Alcohol Survey (CTDAS)	Canada	20-24	1	2013-2017	GP		LT, 1Y		Telephone CATI	2013: 81.8%[b]<br>2017: 37.5%[b]
Irish Health Survey (IHS)	Ireland	25-34	1	2015-2019	GP		1Y		2015: Postal SAPQ<br>2019: In person CAPI	Unclear[d]
Irish National Drug and Alcohol Survey (INDAS)	Ireland	25-34	1	2002/3-2019/20	GP			1Y	In person CAPI	2002: 70%[b]<br>2019/20: 63.9%[b]
New Zealand Health Survey (NZHS)	New Zealand	18-34	1	2006/7-2018/9		GP	1Y		In person CAPI	2006/7: 67.9%[b]<br>2018/19: 80%[b]

New Zealand National Alcohol Survey (NZNAS)	New Zealand	18-29	1	2000-2004		GP	1Y		Telephone CATI	2000: 73%[b]<br>2004: 59%[b]

Abbreviations used: M = male, F = female, cons = prevalence of consumption, abs = prevalence of abstention, C = university/college, G = general population, 1Y = past-year, 1M = past-month, 1W = past week, LT = lifetime, NWD = nowadays, US = United States of America, UK = United Kingdom, GB = Great Britain, NI =Northern Ireland

Survey mode abbreviations: ACASI = audio-computer-assisted self-interview, CAPI = computer-assisted personal interviewing, CASI = computer=assisted self-interviewing, CATI = computer-assisted telephone interview, CAWI = computer-assisted web interview, F2F (NOS) = face to face interview (method not otherwise specified), PAPI = pen and paper interview (with interviewer present), SAPQ = self-administered paper questionnaire

[a]The sixteen records from different waves of seven surveys are included as one longitudinal record per survey, with the data from the more recent record considered here for 'source' purposes.

[b]Response rate for whole survey, rather than the age group specified in this review.

[c]Monitoring the Future consists of a cross-sectional survey in 12th grade, with participants then selected to continue in a "panel" survey. This therefore has both cross-sectional and cohort elements.

[d]Author unable to determine the response rate based on material in the public domain.

Sources of further methodological information about each survey are available in the table below.

Survey	Information Source
Behavioral Risk Factor Surveillance System (BRFSS)	https://www.cdc.gov/brfss/publications/index.htm<br>https://www.cdc.gov/brfss/annual_data/2019/pdf/2019-sdqr-508.pdf (most recent)
Monitoring the Future (MTF)	http://monitoringthefuture.org/pubs.html
National Alcohol Survey (NAS)	https://arg.org/center/national-alcohol-surveys/<br>https://arg.org/research/methodology/
National Epidemiological Survey on Alcohol and Related Conditions (NESARC)	https://www.niaaa.nih.gov/research/nesarc-iii/methodology
National Health Interview Survey (NHIS)	https://www.cdc.gov/nchs/nhis/methods.htm
National Health and Nutrition Examination Survey (NHANES)	https://wwwn.cdc.gov/nchs/nhanes/analyticguidelines.aspx
National Survey on Drug Use and Health (NSDUH)	https://www.samhsa.gov/data/report/nsduh-2019-methodological-resource-book-mrb (most recent)
General Household Survey (GHS)	https://www.ons.gov.uk/peoplepopulationandcommunity/housing/methodologies/integratedhouseholdsurveyqmi (most recent - General Household Survey part of the Integrated Household Survey since 2008)<br>https://webarchive.nationalarchives.gov.uk/ukgwa/20160108004349/http://www.ons.gov.uk/ons/rel/ghs/general-household-survey/index.html
Health Survey for England (HSE)	https://files.digital.nhs.uk/CA/2393EF/HSE18-Methods-rep.pdf (most recent)<br>https://files.digital.nhs.uk/4D/6201E1/HSE18-Data-Quality-v3.pdf (most recent)<br>https://files.digital.nhs.uk/7E/19016E/HSE18-Survey-Documentation-rep.pdf (most recent)
Opinion and Lifestyle Survey (OLS)	https://www.ons.gov.uk/peoplepopulationandcommunity/healthandsocialcare/healthandlifeexpectancies/methodologies/opinionsandlifestylesurveyqmi<br>https://www.ons.gov.uk/aboutus/whatwedo/paidservices/opinions/opinionsandlifestylesurveymethodology
Scottish Health Survey (SHeS)	https://www.gov.scot/publications/scottish-health-survey-2019-volume-2-technical-report/ (most recent)

National Survey for Wales (NSW)	https://gov.wales/national-survey-wales-technical-information
Adult Drinking Patterns in Northern Ireland (ADPNI)	https://www.health-ni.gov.uk/sites/default/files/publications/dhssps/adps-2013.pdf (most recent)
Continuous Household Survey (CHS)	https://www.nisra.gov.uk/statistics/find-your-survey/continuous-household-survey
Australian National Health Survey (ANHS)	https://www.abs.gov.au/methodologies/national-health-survey-first-results-methodology/2017-18 (most recent)
National Drug Strategy Household Survey (NDSHS)	https://www.aihw.gov.au/about-our-data/our-data-collections/national-drug-strategy-household-survey/2019-ndshs (most recent) https://meteor.aihw.gov.au/content/index.phtml/itemId/730155 (most recent)
Canadian Alcohol and Drug Use Monitoring Survey (CADUMS)	https://www.canada.ca/en/health-canada/services/health-concerns/drug-prevention-treatment/canadian-alcohol-drug-use-monitoring-survey.html
Canadian Tobacco, Drugs and Alcohol Survey (CTDAS)	https://www23.statcan.gc.ca/imdb/p2SV.pl?Function=getSurvey&SDDS=4440#a3 (most recent)
Irish Health Survey (IHS)	https://www.cso.ie/en/methods/surveybackgroundnotes/irishhealthsurveyihs/
Irish National Drug and Alcohol Survey (NDAS)	https://www.hrb.ie/fileadmin/2._Plugin_related_files/Publications/2021_publications/2021_HIE/Evidence_Centre/The_2019-20_Irish_National_Drug_and_Alcohol_Survey._Main_findings.pdf (see Chapter 2)
New Zealand Health Survey (NZHS)	https://www.health.govt.nz/nz-health-statistics/national-collections-and-surveys/surveys/new-zealand-health-survey#method https://www.health.govt.nz/publication/methodology-report-2019-20-new-zealand-health-survey (most recent)
New Zealand National Alcohol Survey (NZNAS)	http://www.aphru.ac.nz/projects/alcohol%202000%20method.htm See also Huckle *et al.*[58]

Supplementary Material 7 – Quality Assessment Table

Study	Survey (if multiple)	Appropriate Sample Frame?	Appropriate Sampling?	Adequate Sample Size?	Sufficient Details of Subjects and Setting?	Sufficient Coverage of Sample?	Valid Methods?	Reliably Conducted?	Appropriate Statistical Analysis?	Adequate Response Rate?	Comments
Adult Drinking Patterns in Northern Ireland, 2005 (report) (Central Survey Unit, 2006)		Y	Y	Y	N	Y	Y	Y	N	Y	18-29 19% sample, 23% population. No numerator and denominator. No confidence intervals for percentages.
Adult Drinking Patterns in Northern Ireland, 2008 (report) (Central Survey Unit, 2008)		Y	Y	Y	N	Y	Y	Y	N	Y	18% sample, 24% population. No numerator and denominator. No confidence intervals for percentages.
Adult Drinking Patterns in Northern Ireland, 2013 (report) (Information Analysis Directorate, 2014)		Y	Y	Y	N	Y	Y	Y	N	Y	18-29 17% sample, 23% population. No numerator and denominator. No confidence intervals for percentages.
Australian National Health Survey, 2001 (report) (ABS, 2002)		Y	Y	Y	N	?	Y	Y	Y	?	Unclear response rate and coverage. Weighted estimates available so classed as providing numerator and denominator, but not classed as providing sufficient details of

										subjects and setting as no sample size available.
Australian National Health Survey, 2017/18 (data sheet) (ABS, 2017-18)		Y	Y	Y	Y	Y	Y	Y	Y	Y	Numerator and denominator reported plus confidence intervals reported. Sample size reported by age and sex
Callinan et al., 2017		Y	Y	Y	Y	?	Y	Y	N	Y	Numerator and denominator not reported. Discussion of response rate noted. Response rate broken down by age and sex – different age bands used in for different survey years so difficult to directly compare, but coverage appears similar to other surveys (~5 percentage points less than population estimate).

Canadian Alcohol and Drug Use Monitoring Survey (CADUMS)/ Canadian Tobacco, Alcohol and Drugs Survey (CTADS) (Government of Canada, 2020)	CADUMS	Y	Y	Y	N	?	Y	Y	N	?	Numerator and denominator not reported. Unclear response rate for CADUMS 2008.
	CTADS	Y	Y	Y	N	?	Y	Y	N	Y	No numerator and denominator. Unclear coverage in 20-24 age group.
Canadian Tobacco, Alcohol and Drugs Survey, 2013 (data sheet) (Government of Canada, 2016)		Y	Y	Y	N	?	Y	Y	Y	Y	Sample size and percentage with confidence intervals reported. Unclear coverage in 20-24 age group.
Canadian Tobacco, Alcohol and Drugs Survey, 2017 (data sheet) (Government of Canada, 2021)		Y	Y	Y	N	?	Y	Y	N	?	No numerator and denominator, though percentages presented with confidence intervals. Unclear coverage in 20-24 age group. Response rate of 37.5% with no further detail in the public domain.
Chen & Yoon, 2021		Y	Y	Y	N	?	Y	Y	N	Y	No numerator and denominator. Unclear coverage of 18-20 age group.
Continuous Household Survey		Y	Y	Y	N	?	Y	Y	N	?	Bases reported for 2008/9 only. Unclear response

(dhata sheet) (NISRA, 2017)											rate and overall coverage of 20-24 age group.
Dawson et al., 2015		Y	Y	Y	N	?	Y	Y	N	Y	Good coverage of age group by sex in NESARC-III but not reported for NESARC-1 (though oversamples 18-24s). No numerator and denominator.
Grant et al., 2017		Y	Y	Y	N	?	Y	Y	N	Y	Good coverage of age group by sex in NESARC-III but not reported for NESARC-1 (though oversamples 18-24s). No numerator and denominator.
Grucza et al., 2018	BRFSS	Y	Y	Y	N	?	Y	Y	N	Y	Unclear young adult coverage. No breakdown of participants other than by state in the public domain. Data reported from estimate – no numerator or denominator available.
	NAS	Y	Y	Y	N	?	Y	Y	N	?	Unclear response rate. Unable to obtain breakdown of sample by age. Data reported from

											estimate – no numerator or denominator available.
	NESARC	Y	Y	Y	N	?	Y	Y	N	Y	Good coverage of age group by sex in NESARC-III but not reported for NESARC-1 (though oversamples 18-24s). Data reported from estimate – no numerator or denominator available
	NHANES	Y	Y	Y	Y	?	Y	Y	N	Y	Different age bands used in results for those in methodology material, but coverage appears good. Data reported from estimate – no numerator or denominator available.
	NHIS	Y	Y	Y	N	?	Y	Y	N	Y	Coverage not available by age and sex. Data reported from estimate – no numerator or denominator available
	NSDUH	Y	Y	Y	N	?	Y	Y	N	Y	Unclear coverage of age group. Data

											reported from estimate – no numerator or denominator available
Hasin et al., 2019		Y	Y	Y	Y	?	Y	Y	N	Y	Unclear coverage of 18-20 age group. No numerator or denominator.
Health Survey for England (2019 data sheet) (NHS Digital, 2020)		Y	Y	Y	Y	Y	Y	Y	Y	?	16-24s made up 9% of sample, around 13% of the population. Bases available for males and females aged 16-24 for all survey years.<br>Unclear response for some survey years.
Huckle et al., 2011		Y	Y	Y	N	?	Y	Y	N	Y	Authors state matched "fairly well" for age but numbers not given. No numerator or denominator.
Irish Health Survey (2015 report) (Central Statistics Office, 2016)		Y	Y	Y	N	?	Y	Y	N	?	Unclear response rate. Number of respondents not broken down by age. No numerator or denominator.
Irish Health Survey (2019 report) (Central Statistics Office, 2020)		Y	Y	Y	N	?	Y	Y	N	?	Unclear response rate. Number of respondents not broken down by age

											or sex. No numerator or denominator.
Livingston, 2015 (report)		Y	Y	Y	Y	?	Y	Y	N	Y	No clear numerator and denominator. Response rate broken down by age and sex – different age bands used in results for those in methodology material, but coverage appears similar to other surveys (~5 percentage points less than population estimate).
McCabe et al., 2021		Y	Y	Y	?	?	Y	Y	N	Y	Unable to access supplementary table which may have clearer subjects and setting detail and sampling of college students. No numerator or denominator reported in main results.
McKetta & Keyes, 2019		Y	Y	Y	N	?	Y	Y	N	Y	Unclear numerator and denominator. Unclear coverage rate or sex breakdown by age.

Mongan et al., 2021 (report)		Y	Y	Y	N	?	Y	Y	N	Y	No numerator and denominator. Unclear coverage of 25-34-year-olds.
National Drug Strategy Household Survey, 2019a (data explorer) (AIHW, 2020)		Y	Y	Y	Y	?	Y	Y	N	Y	No clear numerator and denominator. Response rate broken down by age and sex – different age bands used in for different survey years so difficult to directly compare, but coverage appears similar to other surveys (~5 percentage points less than population estimate).
National Drug Strategy Household Survey, 2019b (data sheet) (AIHW, 2021)		Y	Y	Y	Y	?	Y	Y	N	Y	No numerator and denominator. Response rate broken down by age and sex – different age bands used in for different survey years so difficult to directly compare, but coverage appears similar to other surveys (~5 percentage points less than population estimate).

National Survey on Drug Use and Health (report) (SAMHSA, 2020)		Y	Y	Y	N	?	Y	Y	N	Y	No numerator and denominator reported. Unclear coverage of age group.
National Survey for Wales (data sheet) (Murphy, 2021)		Y	Y	Y	N	?	Y	Y	Y / N	Y	Unclear coverage of 18-30 age group. Sample size and confidence intervals provided for overall total result but not for male/female breakdown.
New Zealand Health Survey (data sheet) (Ministry of Health, n.d.a)		Y	Y	Y	N	?	Y	Y	N	Y	No numerator or denominator. Coverage rates around 40-50% for males and 55-60% for females in the 24-35 age group, Not available for age groups in results.
New Zealand Health Survey (data sheet) (Ministry of Health, n.d.b)		Y	Y	Y	N	?	Y	Y	N	Y	
Ng Fat et al., 2018		Y	Y	Y	N	Y	Y	Y	N	Y	No clear numerator and denominator. No sex breakdown by age for “nowadays” result.
Opinion and Lifestyle Survey (data sheet) (Office for National Statistics, 2018)		Y	Y	Y	Y / N	?	Y	Y	Y / N	?	Unclear response rate. Bases available for GB data for 2005 and 2017 (male and female) but not for England alone.
Schoenborn, 2004 (report)		Y	Y	Y	N	?	Y	Y	N	Y	Unclear numerator and denominator.

(Schoenborn et al., 2004)											Unclear coverage rate or sex breakdown by age.
Schoenborn et al., 2013 (report)		Y	Y	Y	N	?	Y	Y	N	Y	
Schulenberg et al., 2020 (report)		Y	Y	Y	N	Y	Y	Y	N	Y	Unclear numerator and denominator. Majority of the MtF panel is 19-30 so good coverage. Discussion of attrition rates over time.
Scottish Health Survey, 2003 (data sheet) (Bromley et al., 2008)		Y	Y	Y	Y	?	Y	Y	Y	Y	Bases available by age and sex. Unclear coverage of age group.
Scottish Health Survey, 2008 (data sheet) (The Scottish Government, 2009)		Y	Y	Y	Y	Y	Y	Y	Y	Y	Coverage consistent with other samples (3-5% below population estimate depending on sex and age group). Bases available by age and sex.
Scottish Health Survey, 2019 (data sheet) (Scottish Health Survey, 2020)		Y	Y	Y	Y	Y	Y	Y	Y	Y	Coverage consistent with other samples (3-5% below population estimate depending on sex and age group). Bases available by age and sex.
Slater et al., 2015		Y	Y	Y	Y	?	Y	Y	N	Y	Female only dataset. Different age bands

											for coverage used in methodology compared to results. Numerator and denominator not reported.
Smith & Foxcroft, 2009 (report)	CHS	Y	Y	Y	N	?	Y	Y	N	?	No clear numerator and denominator. Unclear response rate and coverage of 20-24 age group.
	GHS	Y	Y	Y	?	?	Y	Y	N	Y	No clear numerator and denominator. Age and sex breakdown provided for 2006 but unable to obtain methodology document for 2000.
Twenge & Park, 2019		Y	Y	Y	N	Y	Y	Y	N	Y	No clear numerator and denominator (figure from graph). Majority of the MtF panel is 19-30 so good coverage – MtF documents discuss attrition rates over time. Unclear sex breakdown by age.
White et al., 2015		Y	Y	Y	N	?	Y	Y	N	Y	No clear numerator and denominator. Response rate broken down by age and sex – different

											age bands used in for different survey years so difficult to directly compare, but coverage appears similar to other surveys (~5 percentage points less than population estimate).

Abbreviations: Y = yes, N = no, ? = unclear. Where survey abbreviations are used, these are consistent with the abbreviations used in the main text and Supplementary Material 5.
